# From Aquatic Pollution to Drinking-Water Exposure: Analytical Challenges in Detecting Nanoplastics in Drinking Water—A PRISMA-Guided Review

**DOI:** 10.3390/molecules31152675

**Published:** 2026-07-31

**Authors:** José Roberto Vega-Baudrit, Mary Lopretti, Felipe Orozco

**Affiliations:** 1 National Nanotechnology Laboratory (LANOTEC), National Center for High Technology (CeNAT), CONARE, San José 10109, Costa Rica; felipeorozcogutierrez@gmail.com; 2 Chemistry School, National University, Heredia 40101, Costa Rica; 3 Laboratory of Nuclear Techniques Applied to Biochemistry and Biotechnology, Nuclear Research Center, Faculty of Sciences, University of the Republic (UDELAR), Mataojo 2055, Montevideo 11400, Uruguay; mlopretti@gmail.com

**Keywords:** nanoplastics, drinking water, bottled water, Raman spectroscopy, SERS, AFM-IR, O-PTIR, Py-GC/MS, analytical chemistry, metrology, PRISMA

## Abstract

Nanoplastics (NPs) in drinking water should be interpreted as the downstream analytical endpoint of a broader continuum of aquatic plastic pollution rather than as an isolated problem. Their detection remains analytically immature because environmentally relevant concentrations are low, particle chemistries are heterogeneous, natural colloids and treatment residuals interfere with measurement, and no single method can simultaneously resolve size, morphology, polymer identity, and mass concentration. Unlike occurrence-centered reviews, this PRISMA-guided review treats drinking-water nanoplastics as a metrological and molecular-identification problem in which preprocessing, particle-level confirmation, polymer-specific quantification, and uncertainty reporting must be integrated. A formal search was closed on 11 April 2026 using prespecified query families across publicly accessible scholarly records and backward citation chaining; 33 unique records were screened, 25 full texts were assessed, and 22 studies were included in the qualitative synthesis. Current evidence indicates that conventional FTIR and routine Raman workflows are inadequate for true nanoscale analysis, whereas advanced Raman-based approaches, AFM-IR, optical photothermal infrared spectroscopy, surface-enhanced Raman spectroscopy, and pyrolysis-gas chromatography-mass spectrometry offer complementary strengths but still have major limitations in throughput, particle-level information, or quantification. The main conclusion is that current uncertainty reflects unresolved analytical chemistry and metrological constraints as much as environmental variability. Regulatory progress will depend on orthogonal workflows, contamination-controlled preprocessing, validated reference materials, LOD/LOQ reporting, and interlaboratory harmonization.

## 1. Introduction

Plastic pollution in drinking water is not an isolated technical issue; it is the final stage of a broader pathway of aquatic contamination that begins with the production, use, abrasion, weathering, and fragmentation of plastic materials in inland and marine environments. Over the last two decades, research on microplastics has shown that fragmented polymers move through rivers, estuaries, groundwater-influenced systems, bottled-water supply chains, and treatment infrastructure before reaching human exposure routes. Within that continuum, nanoplastics (NPs), typically defined operationally as particles below 1 µm, are of particular concern because their small size increases their surface area, mobility, and the likelihood that conventional monitoring workflows will miss them entirely [1,2,3,4].

The drinking-water literature remains analytically fragile. The World Health Organization (WHO) concluded in 2019 that evidence on microplastics in drinking water was limited, methodologically inconsistent, and insufficient for robust health-based assessment, particularly for the smallest particles. WHO’s 2022 follow-up on dietary and inhalation exposures reinforced that the characterization and quantification of nano- and microplastics remain major obstacles to exposure assessment and risk interpretation [1,2]. These conclusions remain highly relevant because nanoplastics fall precisely within the size range where concentration estimates are most method-dependent.

The central problem is measurement science. The U.S. Environmental Protection Agency (EPA) states that no standalone method can characterize the broad diversity of micro- and nanoplastic particles encountered across environmental matrices and emphasizes the need to standardize methods for collection, extraction, quantification, and identification [5]. NIST similarly notes that current methods commonly used for microplastics generally lack the sensitivity needed for nanoplastics, and that available test materials are insufficient to support the metrology required for regulatory and industrial use [6,7]. In practical terms, the field is attempting to quantify an analyte that is operationally defined by methods that are still under development.

From an analytical chemistry perspective, NPs are not a single conventional analyte but a polydisperse particulate distribution whose reported concentration depends on operational size cutoffs, extraction chemistry, surface adsorption losses, spectral library matching, polymer marker selection, calibration strategy, and blank correction. Consequently, a defensible drinking-water result must connect four elements: a defined nanoscale fraction, polymer-specific molecular evidence, a quantitative endpoint with stated uncertainty, and a QA/QC framework capable of distinguishing true plastic signals from natural colloids, additives, degradation products, and laboratory background.

This difficulty is magnified in drinking-water-related matrices. Tap water is comparatively clean relative to wastewater or sediments, but it still contains inorganic colloids, natural organic matter, residual treatment chemicals, packaging-derived particles, and laboratory contamination risks that can distort nanoscale measurements. The emerging primary literature shows that nanoplastic-like signals have been reported in tap, bottled, and drinking water, and at potable water treatment plants, yet the numerical outputs vary not only because the environments differ but also because the workflows define the measurable particle population differently [8,9,10,11,12,13]. Some studies report mass concentration; others emphasize mean size, hydrodynamic diameter, particle abundance, or polymer fingerprints. Those endpoints should not be conflated.

This matters beyond academic precision. If the field cannot determine whether the most defensible endpoint is polymer-specific mass, particle count, size distribution, or particle-resolved chemical identity, then exposure estimates remain difficult to compare across studies and nearly impossible to translate into consistent monitoring strategies. The scientific contribution, therefore, lies less in repeating that nanoplastics may occur in drinking water and more in critically assessing how aquatic plastic contamination becomes measurable, or fails to become measurable, within potable-water contexts.

The aims of this review are fourfold: first, to frame drinking-water nanoplastics as a downstream expression of aquatic plastic pollution; second, to evaluate the analytical workflows currently used to detect, identify, and quantify nanoplastics in drinking-water-related matrices; third, to examine the main sources of uncertainty, especially those arising from sample preparation and metrological limitations; and fourth, to identify the methodological priorities most likely to move the field from proof-of-concept detection toward regulatory readiness [1,2,5,6,7,14,15].

## 2. Review Design and Methodological Framework

This manuscript is structured as a PRISMA-guided review rather than as a pooled quantitative meta-analysis. PRISMA 2020 was used as the reporting framework because the topic requires transparency in search strategy, study selection, and evidence synthesis; however, a formal meta-analysis is not yet appropriate for most drinking-water nanoplastics studies, as reported outcomes remain fundamentally heterogeneous. Across the literature, endpoints include particle counts, hydrodynamic diameters, mean particle sizes, polymer-specific mass concentrations, and qualitative particle identification. Combining such outputs statistically would create an artificial precision that the underlying measurements do not support [14,15].

No formal review protocol was registered. However, the search strategy, eligibility criteria, screening log, extraction fields, and method-centered quality appraisal framework were defined prior to qualitative synthesis and are provided in Supplementary File S1 to support transparency, auditability, and reproducibility.

The formal search was closed on 11 April 2026. To keep the evidence base fully auditable within the manuscript-preparation workflow, the search was deliberately restricted to publicly accessible scholarly records, specifically PubMed-indexed records, publisher landing pages, and backward citation chaining from key primary papers. Four prespecified query families were used to capture (i) drinking-water occurrence studies, (ii) bottled-water and treatment-plant studies, (iii) transferable nanoplastics methods in aqueous matrices, and (iv) hybrid single-particle platforms with explicit water relevance. This approach was judged preferable to report unverifiable counts from subscription-gated export tools.

Eligibility criteria retained original peer-reviewed studies that described the detection, identification, or quantification of nanoplastics in tap water, bottled water, potable waters, drinking-water treatment systems, or directly transferable water matrices. Records were excluded when they were reviews, reports, editorials, microplastics-only studies without nanoscale analytical claims, or surrogate-removal studies that did not analytically detect environmental or quasi-environmental nanoplastics. Pure toxicology papers were also excluded because the review objective was analytical capability rather than hazard synthesis.

**Figure 1:**
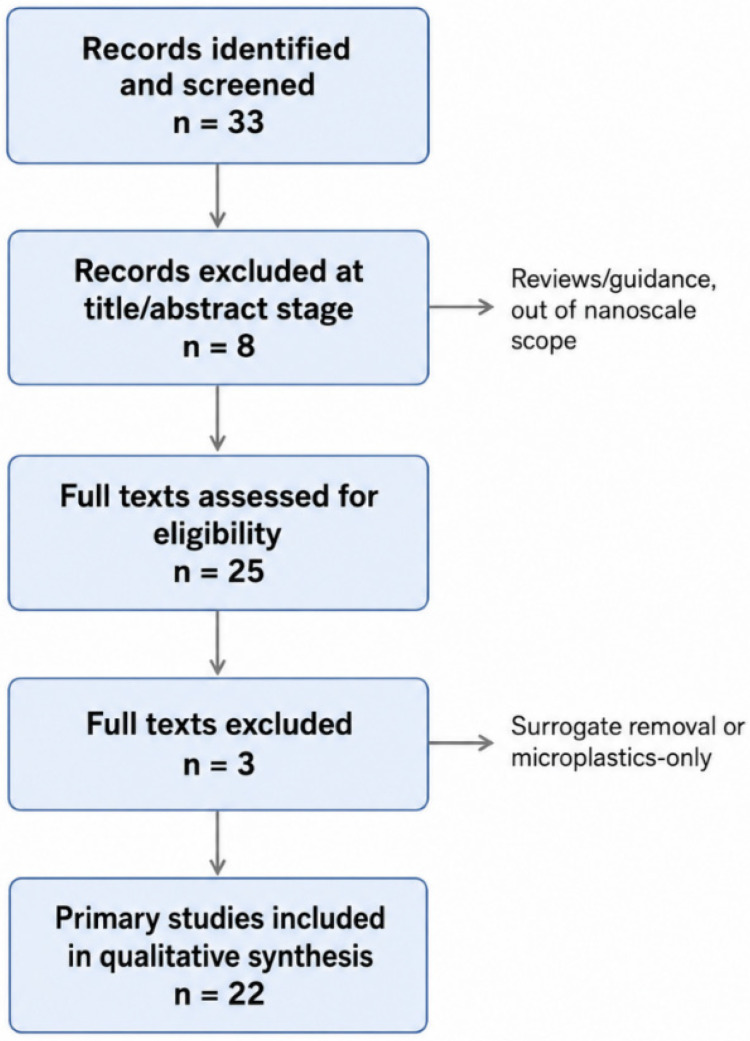
PRISMA-style flow diagram for the closed formal search and final study selection. The value n = 22 refers only to the studies included in the qualitative synthesis; additional contextual and metrological references cited in the discussion were not part of this count.

The data extraction extended beyond descriptive study characteristics to include a comparative QA/QC and validation layer. Specifically, each included study was coded for blank/contamination control, recovery or trueness evidence, calibration or LOD/LOQ reporting, polymer-confirmation route, endpoint class, and the principal reliability constraint. When a parameter was not explicitly reported or could not be inferred from the analytical design, it was coded as NR rather than imputed. This conservative coding is important because absence of reporting is itself a metrological finding in drinking-water nanoplastic analysis. Records were captured and deduplicated in real time by title and DOI. The closed search yielded 33 unique records. After title/abstract screening, 25 full texts were assessed for eligibility, and 22 studies were included in the final qualitative synthesis. Eight records were excluded at the title/abstract stage because they were reviews, guidance documents, or outside the nanoscale analytical scope; three full-text reports were excluded because they focused on surrogate-removal experiments or microplastics-only monitoring rather than analytical detection of environmental nanoplastics. Figure 1 summarizes the flow, and Section 8 provides the closed search audit and screening log.

For each included study, the extraction framework recorded matrix type, sample volume, contamination control measures, pretreatment and preconcentration strategy, lower particle-size boundary, polymer confirmation route, reporting metric, and any stated limit of detection or quantification. Emphasis was placed on orthogonal confirmation because claims of nanoplastic occurrence are materially stronger when at least two independent analytical modes converge on the same interpretation. A workflow that pairs particle-resolved spectroscopy with polymer-specific mass spectrometry is therefore more defensible than one based only on light scattering or hydrodynamic sizing [5,6,7,11,12,16].

Quality appraisal in this field must be method-centered. Four questions were prioritized: were blanks reported and interpreted; did sample treatment risk loss, aggregation, fragmentation, or adsorption; was the reported endpoint appropriate for the claim made; and were size boundaries and polymer classes defined in a way that permits comparison across studies? EPA and NIST have both emphasized that reproducible separation, extraction, and characterization protocols are prerequisites for reliable assessment of nanoplastics, so these criteria are not ancillary but the scientific backbone of the present review [5,6,7].

Because the direct occurrence of literature in drinking-water matrices is still comparatively small, the closed PRISMA study set was kept deliberately strict, while a second ring of supporting literature was incorporated to strengthen interpretation of sampling, quality assurance, and quality control (QA/QC), reference materials, metrology, and emerging analytical platforms.

The PRISMA count of n = 22 refers only to the studies included in the qualitative synthesis (8 direct drinking-water studies and 14 directly transferable water-matrix method studies). Additionally, contextual, traceability-based, and best-practice references cited in the discussion were not included in the study count and are provided to support interpretation rather than tabulation of occurrences.

## 3. From Aquatic Pollution to Potable-Water Exposure

A useful starting point is to reject the false separation between “marine plastic pollution” and “drinking-water contamination.” Environmental plastics circulate across connected compartments. Fragmentation in rivers, estuaries, coastal waters, sediments, soils, and engineered systems generates smaller particles that can later re-enter source waters used for drinking-water abstraction. WHO’s 2019 assessment outlined multiple entry routes of plastic particles into drinking-water sources, including runoff, wastewater effluent, industrial discharges, degraded plastic waste, and atmospheric deposition [1]. The broader synthesis of twenty years of microplastic research likewise shows that fragmentation of legacy plastic items continues even when new emissions are reduced, meaning that secondary particles remain a long-term source to aquatic systems [4].

From the standpoint of exposure, the drinking-water segment is therefore both environmentally and technologically mediated. Source-water contamination is only one part of the story. Treatment processes can remove, transform, or redistribute particles; conveyance systems can contribute additional material through abrasion or deposits; and packaging can introduce new particles after treatment. This is especially relevant for bottled water, where the container, cap, liners, and filtration infrastructure may all influence the final particle burden [1,9,10,13]. Consequently, a review limited to “raw occurrence” without considering infrastructure and packaging would systematically underestimate the complexity of potable-water exposure.

The existing literature supports this broader framing. Huang et al. reported the presence of nanoscale organic particles in bottled drinking water and identified bottle degradation as a likely source [9]. Zhang et al. later identified PET nanoplastics in commercially bottled drinking water using SERS, directly linking the detected polymer to packaging-relevant material [10]. Li et al. demonstrated the presence of PE and PVC nanoplastics along a drinking-water treatment train and suggested that an ozonation contact tank may function as a local source or transformation zone for nanoscale plastic material [12]. Hart and Lenhart recently emphasized that the occurrence of micro- and nanoplastics in treated and bottled drinking water is still poorly understood, largely because available methods remain limited [13]. These studies point in the same direction: potable-water exposure cannot be separated from the aquatic and engineered systems that precede the point of consumption.

The analytical consequence of this continuum is that the matrix varies continuously along the pathway from the environmental source to the consumer product. River-derived waters contain natural colloids, mineral particles, and fluctuating organic matter. Treated waters may have lower particulate burden but may also contain residual treatment chemicals or altered particle-size distributions. Bottled waters add packaging-specific contributions and potentially new background matrices. A method that performs adequately in one segment of this continuum may fail in another. For that reason, analytical validation in model suspensions or clean laboratory water is informative, but not sufficient; real-world transferability must be demonstrated across the relevant aquatic-to-potable pathway [5,7,17,18].

This continuum perspective clarifies the scientific relevance of the topic. The novelty is not that a drinking-water plant contains pumps, membranes, or ozone. The novelty is that aquatic plastic pollution migrates through those systems to an exposure-relevant endpoint, and that current analytical methods still struggle to describe that transfer with defensible precision. In other words, the scientific target is not water-treatment engineering by itself, but the metrology of a pollution signal as it moves from aquatic environments to human intake.

**Figure 2:**
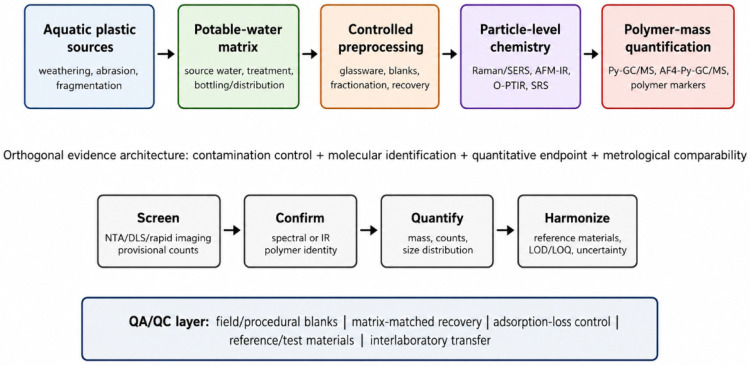
Conceptual analytical-chemistry continuum linking aquatic plastic inputs, potable-water matrix formation, contamination-controlled preprocessing, particle-resolved molecular identification, polymer-specific mass quantification, and metrological harmonization. The figure emphasizes that the reported drinking-water nanoplastic signal is produced by the entire workflow, not by the detector alone.

Figure 2 operationalizes the central thesis of this review: the relevant scientific object is not merely a particle in water but a measurement chain. Source-water particles, treatment- or packaging-derived fragments, and laboratory background can converge in the same nanoscale fraction; therefore, analytical confidence requires a workflow that first controls contamination and particle losses, then assigns polymer identity at the particle level, and finally anchors interpretation through polymer-specific mass or size-resolved quantitative endpoints.

## 4. Analytical Platforms: What They Measure, What They Miss, and Why That Matters

### 4.1. Sampling, Preconcentration, and Size Fractionation

The analytical workflow begins long before any detector is switched on. In drinking-water nanoplastics research, sample preparation is not a neutral preprocessing step; it determines the particle population available for measurement. Membrane filtration, ultrafiltration, oxidative digestion, centrifugation, evaporation, and transfer between vessels can all change the apparent abundance and size distribution of the target particles. EPA explicitly identifies the need to separate plastics from organic and inorganic contaminants while avoiding harsh extraction conditions that further degrade the sample [5]. NIST likewise frames size-based separation from complex matrices as a central metrological challenge [7].

The practical literature illustrates why. Li et al. first removed particles larger than 0.45 µm, then fractionated the filtrate using 200, 100, and 20 nm membranes to isolate tap-water nanoplastics [8]. Huang et al. concentrated bottle-derived nanoscale particles and characterized their size distribution using nanoparticle tracking analysis [9]. Okoffo and Thomas combined hydrogen peroxide digestion with ultrafiltration before Py-GC/MS quantification of selected polymer classes in environmental and potable waters [11]. Xu et al. used a similar ultrafiltration-plus-digestion strategy for surface water and groundwater, demonstrating that pretreatment choices can shape the recoverable polymer fraction and the final quantitative result [16]. These workflows represent different analytical endpoints. Each one imposes a different operational definition of what counts as a nanoplastic particle.

The consequences are substantial. Filters may exclude deformable aggregates or retain particles differently depending on surface chemistry. Ultrafiltration can enrich a broad colloidal fraction, including non-plastic material, unless downstream confirmation is rigorous. Oxidative digestion can reduce natural organic matter but may also alter surface signatures or promote aggregation. Even a simple transfer between glass and polymer vessels can produce wall losses at the nanoscale. For this reason, sample preparation uncertainty often exceeds instrumental uncertainty, yet it is frequently underreported. Any serious comparison among studies must therefore begin by asking how the sample was reduced from liters of real water to a small analytical aliquot and what may have been lost, concentrated, or transformed on the way [5,7,11,16].

Recent methodological papers reinforce that this is not a minor procedural issue. Reviews focused on environmental nanoplastic sampling and sample processing emphasize that isolation strategy, colloid carryover, centrifugation conditions, and membrane chemistry can alter the apparent particle population before detection begins [19,20,21].

Practical enrichment studies have shown that ultracentrifugation can recover environmental nanoplastics from water with good separation efficiency, while mini-extruder filtration and related controlled-size workflows help standardize suspensions for downstream benchmarking [22,23]. Best-practice guidance for clean waters further stresses that sample-processing steps must be evaluated as part of the measurement system rather than treated as neutral preparation [24].

### 4.2. Raman Microscopy and Raman Mapping

Raman-based methods remain attractive because they offer chemically specific, non-destructive identification and can operate in water-compatible settings. Their importance in nanoplastics research is clear: when analysts need particle-level chemical information rather than only bulk polymer mass, Raman techniques are often the first option considered. The problem is that conventional Raman microscopy faces the same diffraction-limited spatial constraints that complicate other optical techniques, and the signal quality deteriorates as particle size decreases and matrix complexity increases.

Sobhani et al. provided one of the foundational demonstrations that Raman imaging can visualize and identify microplastics and nanoplastics down to 100 nm under carefully controlled conditions [25]. Fang et al. then showed that Raman imaging could be pushed below the nominal diffraction-limited particle size by careful interpretation of signal distribution and image resolution [26]. These studies were methodologically important because they made explicit the role of laser spot size, particle position within the spot, image resolution, and signal intensity. The message was not that Raman had solved nanoplastic analysis, but that, under optimized conditions and with careful interpretation, it could probe the nanoscale more deeply than conventional workflows were often assumed to allow. In later work on environmentally relevant water matrices, Caldwell et al. tested submicron- and nanoscale plastics spiked into freshwater and saltwater matrices and showed that detection with Raman spectroscopy is strongly matrix-dependent [17]. That is a critical insight because a method that performs well in purified suspensions may behave differently in environmental or potable water containing background particulates and dissolved material.

Raman’s strengths are therefore real but conditional. It offers polymer fingerprinting, preserves particle-level context, and is compatible with integrated microscopy workflows. At the same time, Raman throughput is low for sparse environmental samples, fluorescence can obscure spectra, and the smallest particles may yield ambiguous or weak signatures. For drinking-water analysis, Raman is best interpreted as a high-specificity component within a cross-validated workflow rather than as a universal stand-alone solution [17,25].

#### Reliability Limits of Raman Identification in Nanoscale Water Matrices

A critical distinction is required between chemical specificity under optimized optical conditions and reliability in real water matrices. For nanoplastics, Raman spectra may be compromised by fluorescence from natural organic matter, weak scattering from particles smaller than or comparable to the laser spot, poor particle centering within the focal volume, local heating, baseline drift, cosmic-ray removal routines, and spectral library mismatch. These effects do not merely reduce cosmetic spectral quality; they alter the probability of false negatives when true plastic particles yield weak spectra and false positives when partial bands are overmatched to polymer libraries. Therefore, a Raman-based occurrence claim is most defensible when the authors report background subtraction rules, match thresholds, rejected spectra, blank spectra, particle-size constraints, and an orthogonal confirmation route [17,24,25,26,27,28,29].

Reproducibility is also a sampling problem, not only an instrumental problem. Sparse nanoparticles deposited on a filter or substrate are not distributed as a perfectly homogeneous analytical film. Different mapped areas, focusing conditions, integration times, objective numerical apertures, and preprocessing pipelines may produce different apparent particle populations from the same sample. For that reason, Raman microscopy should be reported with acquisition parameters, mapping area, spectral decision rules, and replicate strategy. Without these details, the method may still demonstrate feasibility, but it cannot support robust interlaboratory comparability.

### 4.3. Surface-Enhanced Raman Spectroscopy and Related Enhanced Optical Approaches

Surface-enhanced Raman spectroscopy (SERS) has become one of the most promising families of methods for waterborne nanoplastic analysis because it addresses Raman’s central weakness: sensitivity. By exploiting plasmonic amplification at metallic nanostructured surfaces or colloids, SERS can greatly increase the signal intensity for low-mass, small-diameter particles in aqueous media. Lv et al. established early feasibility for aquatic matrices [30], Chaisrikhwun et al. reported size-independent quantification across 100–800 nm aqueous media [31], Ruan et al. extended rapid detection down to 20 nm in water [32], and Luo et al. showed that ring-shaped nanogap arrays can detect 50 nm polystyrene in minimal sample volumes [33]. Shorny et al. further demonstrated single-particle and agglomerate imaging down to 100 nm [34]. These studies suggest that enhanced optical routes can meaningfully extend detection into the nanoscale, but they also remain substrate- and calibration-sensitive.

Lv et al. evaluated in situ SERS for micro- and nanoplastics in aquatic environments, showing the conceptual feasibility of enhanced Raman approaches for water analysis [30]. Chaisrikhwun et al. tackled a specific quantification problem by reducing the size-dependent signal bias that complicates SERS-based analysis, enabling quantification of polystyrene nanospheres of different diameters in various aqueous media [31]. Ruan et al. pushed the size boundary further by reporting rapid SERS-based detection of nanoplastics as small as 20 nm in water [32]. Zhang et al. used SERS to identify PET nanoplastics in commercially bottled drinking water, a particularly important application because it linked a high-sensitivity optical method to a real consumption matrix rather than a model suspension [10].

Enhanced Raman methods, therefore, occupy a strategically important space in the analytical landscape. They can approach the small-size sensitivity needed for nanoplastics while retaining chemical specificity. However, that strength comes with constraints. Signal intensity depends on substrate properties, particle-substrate interactions, aggregation state, and calibration strategy. Cross-laboratory comparability remains weak because substrates, sample deposition routes, and data-processing pipelines vary. SERS can be genuinely powerful, but it is not yet standardized enough to function as a universal monitoring method for drinking-water regulation [10,30,31,32].

#### SERS Sensitivity, Optical Artifacts, and Calibration Dependence

SERS improves sensitivity but introduces a different reliability problem: the analytical signal becomes a function of the plasmonic substrate as well as of the particle. Enhancement factors depend on nanogap geometry, hot-spot distribution, particle-surface contact, colloid aggregation, deposition uniformity, laser polarization, and the local chemical environment. A high SERS signal is therefore not automatically equivalent to a robust concentration estimate. Quantification requires polymer-specific calibration under substrate-, deposition-, and matrix-matched conditions; otherwise, apparent sensitivity may conceal poor trueness and weak reproducibility [10,30,31,32,33,34,35].

The most important practical risk is that SERS can amplify both the target signal and matrix-derived spectral artifacts. Natural organic matter, surfactants, salts, nanometals, container-derived organics, and incompletely removed additives may compete for active sites or modify particle adsorption. Consequently, SERS studies should report substrate characterization, blank spectra, negative controls, matrix-matched calibrants, replicate substrates, spectral-selection thresholds, and the procedure used to distinguish single particles from agglomerates. These reporting elements are necessary for evaluating sensitivity, reproducibility, accuracy/trueness, and robustness rather than only nominal particle-size reach.

A closely related advance is stimulated Raman scattering (SRS) microscopy. Qian et al. reported rapid single-particle chemical imaging of nanoplastics using SRS microscopy, describing high sensitivity and chemical specificity, with far greater imaging speed than conventional Raman mapping [18]. A different but equally important direction is AI-assisted nanodigital in-line holographic microscopy: Wang et al. reported real-time in situ physicochemical characterization and automated detection of nano- and microplastics in aquatic systems [36]. These are major conceptual steps because they address the long-standing trade-off between throughput and chemical confidence. Although SRS and AI-assisted holographic platforms are currently specialized rather than routine, they illustrate the direction the field is moving toward fast, particle-resolved, chemically informed imaging that can interrogate large numbers of very small particles without surrendering analytical specificity.

Recent technical reviews confirm that enhanced optical methods are evolving from proof-of-concept tools into a broader analytical family that includes SERS, advanced Raman imaging, machine-learning-assisted spectral interpretation, and fast chemical imaging platforms [27,28,35,37,38,39]. Their collective value lies not in already offering a universal routine method, but in showing how chemical specificity at progressively smaller size scales can be achieved when optics, substrates, and data analysis are co-optimized [27,28,29].

**Figure 3:**
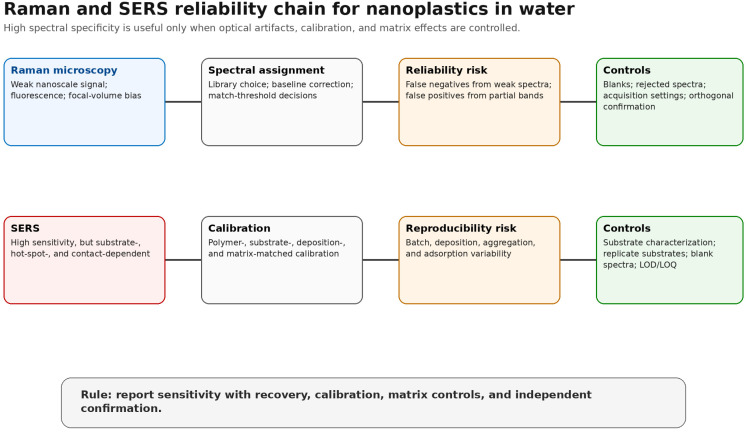
Original schematic showing the main reliability constraints of Raman and SERS for nanoplastics in water. Raman is limited mainly by weak nanoscale scattering, fluorescence, focal-volume effects, and spectral matching; SERS improves sensitivity but adds substrate, hot-spot, deposition, and calibration dependencies. The figure emphasizes that spectral identification is strongest when matrix controls and orthogonal confirmation are included.

Figure 3 summarizes the reliability chain for Raman and SERS workflows. The diagram is original and is not reproduced from the cited papers; it was added to make explicit how optical sensitivity, spectral decision rules, matrix controls, and calibration jointly determine confidence.

### 4.4. FTIR, AFM-IR, and the Infrared Challenge at the Nanoscale

Infrared spectroscopy is indispensable in microplastics research, but its role in nanoplastics is much more complicated. Conventional FTIR is robust for micrometer-scale polymer identification, yet its spatial resolution is poorly matched to true nanoplastic analysis. The problem is structural: standard far-field infrared measurements average over sampling volumes that are simply too large for isolated nanoparticles in complex aqueous matrices. This does not make FTIR irrelevant, but it does make conventional FTIR insufficient as a primary method for the smallest drinking-water particles [5,6,7].

The field’s response has been to move toward nanoscale or near-field infrared approaches. AFM-IR is particularly important because it combines high-spatial-resolution topography with infrared absorption-based chemical information at the single-particle level. Li et al. used AFM-IR together with Py-GC/MS to identify and quantify PE and PVC nanoplastics in the 20–1000 nm range within a drinking-water treatment plant [12]. That study is methodologically influential because it demonstrates how particle-resolved chemical identification and bulk polymer quantification can be combined in a single workflow, reducing the ambiguity that arises when only one analytical perspective is used.

AFM-IR’s advantages are clear. It can chemically interrogate individual particles at a spatial scale inaccessible to routine FTIR, and it is particularly valuable when particle morphology is critical to interpretation. Its main limitations are equally clear: low throughput, demanding sample preparation, specialist instrumentation, and limited practicality for high-volume monitoring. In other words, AFM-IR is closer to a confirmatory or reference-level technique than to a rapid screening platform. Within the current state of the field, its most defensible role is as part of orthogonal method validation rather than as a universal survey tool [7,12].

The broader lesson is that infrared methods remain highly relevant, but only when their scale limitations are acknowledged explicitly. Literature is strongest when authors use high-resolution infrared tools to answer a narrow, chemically specific question and then pair those answers with complementary mass-based or optical information. Literature is weakest when conventional IR methods are asked to support particle-level claims they were never designed to resolve.

Optical photothermal infrared (O-PTIR) spectroscopy and related submicron infrared approaches now deserve explicit attention in this context. Recent tutorial and application literature shows that O-PTIR can narrow the spatial mismatch that limits conventional IR microscopy and can be combined productively with AFM-IR to strengthen submicron polymer identification [40,41,42,43]. For drinking-water questions, the significance of O-PTIR is strategic: it provides an additional orthogonal route for chemically specific interrogation when Raman suffers from fluorescence or when bulk thermal approaches lack particle-resolved context [13,40,42].

### 4.5. Nanoparticle Tracking Analysis, Dynamic Light Scattering, and Scattering-Based Metrics

Nanoparticle tracking analysis (NTA) and dynamic light scattering (DLS) are often attractive to analysts because they are operationally simple and produce intuitive outputs, such as size distributions and particle counts or count proxies. In the context of drinking water, however, their interpretive value is fundamentally constrained by a lack of intrinsic chemical specificity. They are useful for describing colloidal behavior; they are not, by themselves, sufficient to prove that a measured nanoscale population is plastic.

This distinction is critical. Huang et al. used NTA as part of a broader characterization strategy for bottled-water nanoparticles, helping to establish the presence of a nanoscale particulate fraction and to estimate its size characteristics [9]. Used in that way, NTA is informative. The problem arises when scattering or tracking outputs are treated as definitive counts of nanoplastic without orthogonal confirmation. In real drinking-water matrices, natural colloids, organic matter, salt effects, and mixed particle populations can distort hydrodynamic sizing and yield misleading abundance estimates. The instrumental output may be precise in a physical sense while remaining chemically ambiguous.

For that reason, scattering-based methods should be understood as contextual tools, not primary proof of polymer identity. They can support pre-screening, optimization of concentration steps, and evaluation of aggregation or dispersion behavior. They become scientifically weak when deployed in isolation. In the present field, one recurring problem is that an elegant colloid measurement is sometimes discussed as though it were a polymer-specific environmental concentration. That is not a minor semantic issue; it is a category error that can inflate confidence in occurrence claims [5,9].

At the same time, the apparent simplicity of scattering outputs can be misleading. Comparative and multiparameter studies show that hydrodynamic size, particle number, and mass concentration are not directly interchangeable, especially when polydispersity, aggregation, or non-plastic colloids are present [44,45,46]. For that reason, NTA and DLS are most defensible when embedded in a broader workflow that includes polymer-specific confirmation and, where possible, an independent mass-based endpoint [45,46].

### 4.6. Pyrolysis-Gas Chromatography-Mass Spectrometry and Other Thermal Methods

Pyrolysis-gas chromatography-mass spectrometry (Py-GC/MS) solves a different analytical problem from microscopy-based methods. Instead of preserving particle structure, it thermally degrades particles and identifies polymers through characteristic pyrolysis products. Its primary strength is therefore quantitative polymer specificity rather than particle-level morphology. For drinking-water nanoplastics, this is extremely valuable because bulk polymer mass can be measured even when direct particle visualization is difficult.

Xu et al. showed that pretreatment via ultrafiltration and digestion could be coupled with Py-GC/MS to quantify selected nanoplastics in surface water and groundwater [16]. Okoffo and Thomas extended this approach to environmental and potable waters and reported polymer-specific concentrations of several common plastics in the low-microgram-per-liter range [11]. These studies matter because they provide a defensible route to quantitative concentration estimates in matrices where particle imaging alone would be highly uncertain.

The limitation, however, is equally important: Py-GC/MS does not preserve particle size, shape, or count. A chemically correct mass concentration can coexist with uncertainty about how many particles were present, whether they were aggregated, or whether they fell predominantly at the lower or upper end of the nanoplastic range. For that reason, Py-GC/MS is most effective when used to anchor chemical identity and polymer mass in a workflow that includes at least one particle-resolved technique. The combined AFM-IR/Py-GC/MS study by Li et al. is exemplary precisely because it links those two analytical dimensions [11,12,16].

### 4.7. Hybrid and Single-Particle Platforms

The most promising future direction is the emergence of hybrid platforms that deliberately combine complementary strengths. Li et al. proposed SEM-Raman as a route to single-particle analysis, integrating high-resolution morphology with Raman chemical information [47]. Schmidt et al. showed how correlative SEM-Raman microscopy can reveal nanoplastics in complex matrices [48], and Schwaferts et al. coupled field-flow fractionation with Raman microscopy via optical tweezers to connect separation with chemical identification [49]. Qian et al. demonstrated SRS microscopy for rapid single-particle chemical imaging [18], Wang et al. introduced AI-assisted nano-DIHM for real-time in situ detection [36], and Li et al. combined AFM-IR with Py-GC/MS in a treatment-plant setting [12]. These studies share a common logic: no single instrument currently gives a fully satisfactory answer, so the best workflows are those that force independent analytical perspectives to converge.

This is more than a technological trend; it is the field’s clearest answer to its own central paradox. Methods with high chemical specificity often sacrifice spatial or particle-level context, whereas methods with excellent size reach or imaging capability often struggle to achieve unambiguous polymer identification. Hybrid approaches do not magically remove that paradox, but they manage it honestly. For drinking-water nanoplastics, honesty is scientifically preferable to claims of universal method performance that the present evidence cannot justify.

Other emerging complementary routes include mass-spectrometric fingerprinting outside the classic pyrolysis workflow. Matrix-assisted laser desorption/ionization time-of-flight mass spectrometry (MALDI-TOF-MS) has been proposed as a rapid method for identifying nanoplastics and microplastics at small scales, while hybrid data-driven platforms increasingly couple spectral and imaging information to improve particle discrimination [34,46,50]. Although these methods are not yet routine in drinking-water surveillance, they expand the menu of orthogonal confirmation strategies for testing ambiguous results from more established workflows [28,50].

Hybridization is also expanding toward size-resolved composition rather than single-instrument compromise. Recent work integrating asymmetric flow field-flow fractionation (AF4) with Py-GC/MS has shown that polymer composition can be linked more directly to size-resolved fractions, while AI-assisted multidimensional characterization and other integrated platforms point toward future workflows in which particle imaging, chemistry, and quantification are interpreted jointly rather than sequentially [36,46,51]. This direction is consistent with the broader analytical outlook emerging from recent review literature, which increasingly frames nanoplastic detection as a systems problem requiring complementary measurements rather than a search for a single perfect instrument [37,38,39].

Table 1 consolidates the platform-level evidence discussed above and should be read as a decision matrix rather than as a ranking. Each method resolves one analytical dimension while leaving another underconstrained: Raman and SERS strengthen polymer identification but differ in sensitivity and standardization; AFM-IR and O-PTIR improve nanoscale infrared specificity but remain specialized; NTA and DLS are useful mainly as screening tools; and Py-GC/MS provides robust polymer mass while sacrificing particle morphology. The comparison, therefore, operationalizes the central argument of this section: drinking-water nanoplastic analysis is an exercise in complementarity, not in selecting a single fashionable instrument.

The practical implication of Table 1 is that method selection must be guided by the endpoint. A surveillance program focused on temporal trends in polymer mass will naturally privilege Py-GC/MS, whereas a source-apportionment or transformation study will require particle-resolved confirmation. SERS or SRS may be especially useful for bottled-water matrices where small particle size and PET-like signals are central, while AFM-IR or O-PTIR are more defensible as reference-level tools for chemically ambiguous particles. This division of labor prevents a common overstatement: a technique that is strong for one endpoint should not be presented as sufficient for all endpoints.

The practical implications of Table 2 are both editorial and scientific. Manuscripts on drinking-water nanoplastics should not be evaluated only by whether they deploy advanced instrumentation; they should be evaluated by whether the measured signal is traceable from sample collection to polymer-specific confirmation and quantitative reporting. This criterion is especially important for studies in clean waters, where blank control, recovery, and endpoint definition may determine the apparent result more strongly than detector sensitivity.

## 5. Evidence from Drinking-Water-Related Matrices

### 5.1. Tap Water and Potable Waters

The evidence for nanoplastics in tap and potable waters is no longer hypothetical, but it remains strongly method-bound. Li et al. reported nanoplastics in tap water after sequential fractionation and multipronged confirmation, with the most frequent particle sizes in the 58–255 nm range and an estimated abundance of 1.67–2.08 µg/L [8]. This study is often cited because it provided a practical workflow that moved beyond broad suspicion and into sample-specific identification. Its importance is justified. At the same time, the reported concentration is inseparable from the underlying operational choices: cut-off filters, isolation steps, confirmation thresholds, and the assumption that the retained particles remained representative of the original sample. The result is scientifically meaningful, but it is not independent.

Mass-based studies reinforce the plausibility of occurrence. Okoffo and Thomas quantified selected nanoplastic polymers in environmental and potable waters using Py-GC/MS and reported that PE, PET, PP, and PS were prevalent at low µg/L levels [11]. Because their method focused on polymer-specific pyrolysis products, the study provided greater chemical certainty than many particle-count approaches. Yet the gain in quantitative confidence came at the cost of particle-level information. This does not weaken the study; rather, it clarifies what kind of question it answers best. It is excellent for the question “Which polymers, and how much mass?” and less informative for the question “How many particles of what size and shape?” Hart and Lenhart recently compared treated drinking water and bottled water and emphasized that nanoplastic occurrence in drinking water remains poorly understood, primarily because methods to isolate and analyze nanoplastics are still limited [13]. That conclusion is analytically important because it resists the temptation to overread the available evidence. Even where occurrence is plausible, the confidence interval around any absolute concentration remains broad when different studies use incompatible endpoints and size definitions. The present literature, therefore, supports a cautious recognition of exposure potential rather than methodological closure.

### 5.2. Bottled Water and Packaging-Related Contributions

Bottled water provides one of the clearest examples of why the interpretation of exposure must include post-treatment processes. Huang et al. analyzed organic nanoparticles in two commercial bottled waters and identified bottle degradation as a likely source, highlighting that the water product and its packaging cannot be treated as analytically independent [9]. This paper is particularly valuable because it shifts the question from “Are nanoplastics in the water source?” to “How many particle-generating steps exist between source water and consumption?” Zhang et al. later identified PET nanoplastics in commercially bottled drinking water by SERS, reporting a mean particle size of approximately 88.2 nm [10]. This result is analytically consequential for two reasons. First, the identified polymer was directly compatible with bottle-related materials. Second, the study showed that enhanced optical spectroscopy could move beyond idealized laboratory particles and into a real commercial matrix. It therefore functions simultaneously as evidence of occurrence and as a demonstration of method transferability.

The bottled-water literature also underscores an important interpretive caution: high apparent particle burden does not necessarily imply that all particles arise from the same step in the supply chain. Particles may originate from bottle walls, caps, liners, filtration components, transport stress, storage conditions, or even sample handling after purchase. For that reason, bottled-water data should be read as exposure-relevant but mechanistically composite. They reveal what consumers may encounter, but not always which specific stage created the observed burden [9,10,13,18].

### 5.3. Drinking Water Treatment Plants as Removal and Transformation Environments

Treatment systems occupy a scientifically awkward but important position in the literature. On the one hand, they are engineered barriers designed to remove particles. On the other hand, they can also modify plastics through oxidation, abrasion, shear, chemical dosing, or selective retention and breakthrough. Li et al. provided one of the most informative studies in this domain by combining AFM-IR and Py-GC/MS to identify and quantify nanoplastics from 20 to 1000 nm in a drinking-water treatment plant [12]. Their data indicated the presence of PE and PVC nanoplastics and suggested that the ozonation contact tank acted as a source zone or transformation hotspot within the treatment train. Complementary mass-based evidence was later provided by Xu et al., who quantified MPs and NPs across the full treatment train by Py-GC/MS and showed that treated water still contained measurable polymer mass, with lower apparent removal for the smallest fractions [52].

This finding is conceptually important. It implies that treatment systems cannot be assessed solely in terms of net removal; they must also be evaluated for their capacity to alter particle-size distributions, surface chemistry, and polymer-specific abundance. A process that reduces larger particle counts but generates or mobilizes smaller plastic fragments may appear effective on one metric and less effective on another. That ambiguity is one reason why the field needs multiple reporting endpoints rather than a single preferred number [1,12,52].

Complementary laboratory- and pilot-scale experiments with Pd-labeled nanoplastics further show that treatment outcomes depend on particle properties, hydraulic conditions, and process sequence, reinforcing that treatment trains should be interpreted as dynamic transformation environments rather than passive barriers [53].

At the same time, treatment-plant studies must be framed carefully. The relevance here is not treatment engineering for its own sake, but the demonstration that aquatic plastic pollution can be transformed within engineered drinking-water systems in ways that affect downstream human exposure. That framing is consistent with the logic of pollution transport, exposure, and measurement.

### 5.4. What the Current Evidence Really Supports

Taken together, the literature supports three conclusions. First, nanoplastics or nanoplastic-like polymer signals have been reported across multiple drinking-water-relevant matrices, including tap water, bottled water, potable waters, and treatment systems [8,9,10,11,12,13,18,52]. Second, the absolute values reported by different studies cannot yet be combined naively because they reflect different operational definitions and analytical endpoints. Third, the strongest studies are those that pair at least two independent analytical perspectives-for example, particle-resolved spectroscopy together with polymer-specific thermal analysis-because they reduce the risk that a single methodological blind spot dominates the interpretation [10,11,12,16,18,36,47,52].

The field has therefore moved beyond the stage of asking whether nanoplastics are conceivable in drinking water. The more important question now is how to convert plausible detection into a comparable measurement. That shift is methodological rather than rhetorical, and it defines the next phase of research.

To connect the matrix-level evidence with the methodological critique, Table 3 distills the principal drinking-water-relevant studies by matrix, workflow, finding, and interpretive caution. The table is intentionally not a prevalence table; it is a confidence table. Its purpose is to show how evidentiary weight changes when the same broad occurrence claim is supported by scattering, enhanced spectroscopy, AFM-IR, Py-GC/MS, SRS microscopy, O-PTIR, or combinations of these approaches.

Table 3 shows a consistent but still incomplete pattern. Tap water, bottled water, potable waters, and treatment plants all provide credible evidence of nanoplastic occurrence or nanoplastic-like polymer signals, yet none offers a complete exposure map on its own. Bottled-water studies are strongest in consumer relevance and packaging plausibility; treatment-plant studies are strongest in transformation mechanisms; and potable-water mass studies are strongest in polymer-specific quantification. The aggregate contribution is therefore methodological triangulation rather than numerical convergence.

## 6. Metrology, Quality Assurance, and Standardization Priorities

The strongest common message across the official agencies and the primary literature is that metrology is the real bottleneck. EPA states that no standalone technique is applicable to the wide variety of micro- and nanoplastic particles and emphasizes the need for standardized methods for collection, extraction, quantification, and identification [5]. NIST is even more explicit: current methods commonly used for microplastics lack the sensitivity needed for nanoplastics, and available materials remain insufficient for the metrology needed by regulators and laboratories [6,7]. These statements are not background decorations; they define the state of the field.

Several practical consequences follow. First, contamination control must become non-negotiable. Because nanoplastics are expected to occur at very low concentrations and at sizes comparable to common laboratory background particles, procedural blanks, field blanks, transport blanks, and airborne contamination controls are essential. A result reported without a blank context is not merely incomplete; in many cases, it is uninterpretable. This is especially true for bottled-water studies and low-particulate-treated waters, where the environmental signal may be on the same order of magnitude as contamination introduced during sample handling [1,5,7].

Second, recovery experiments should be matrix-matched. Spiking pristine polystyrene spheres into clean laboratory water may be useful during method development, but it is not sufficient to characterize performance in real drinking-water matrices containing dissolved salts, natural organic matter, treatment residuals, and mixed particle populations. Caldwell et al. demonstrated that environmental fresh- and saltwater matrices affect Raman detectability [17]. That lesson almost certainly extends beyond Raman to other size- and surface-sensitive techniques. Method validation in clean media should therefore be regarded as a preliminary step, not final proof of applicability.

In classical analytical chemistry, recovery and trueness are not optional validation ornaments; they determine whether the measured value is an unbiased estimate of the measurand. For nanoplastics, trueness must include losses during vessel contact, filtration, ultrafiltration, digestion, drying/deposition, particle transfer, and detector-specific classification. A spike recovered after only the final instrumental step does not validate the entire method. The preferred design is therefore a full-process, matrix-matched recovery experiment using polymer classes, particle sizes, aging states, and surface chemistries that resemble the intended analytical target.

Third, reporting units must be made more explicit. The current literature mixes at least four fundamentally different analytical outputs: particle counts, particle size statistics, hydrodynamic diameter distributions, and polymer-specific mass concentrations. Each output is legitimate for a different analytical question, but confusion arises when these values are discussed as though they measured the same thing. A polymer mass concentration from Py-GC/MS is not a particle count. A hydrodynamic diameter from NTA is not a chemically confirmed nanoplastic abundance. A robust field will ultimately need workflows capable of delivering more than one endpoint from the same sample or from matched aliquots [5,7,11,16].

Calibration for polymer mass requires the same caution. Py-GC/MS can support relative or absolute polymer-mass quantification only when calibration materials, marker ions, blank subtraction, recovery correction, and uncertainty sources are declared. SERS and other optical methods may also be calibrated, but their calibration functions are typically more substrate- and geometry-dependent than thermal mass calibration. Consequently, calibration should be interpreted as a method-chain property rather than as a detector-only property.

Fourth, reference materials and benchmarking standards are urgently needed. NIST’s metrology program is explicitly focused on test materials, separations, and validated physicochemical characterization protocols because reproducible measurement is impossible without well-characterized controls [6,7]. In drinking-water nanoplastics, the absence of suitable reference materials affects almost every stage of the workflow: recovery estimation, blank correction, matrix-matched calibration, interlaboratory comparison, and method transfer. The field is currently rich in ingenuity but lacks a robust benchmarking infrastructure.

Reference and test materials are now central to progress, not peripheral. Recent work has moved from general calls for standards to practical protocols for producing cry-milled, labeled, and size-controlled micro- and nanoplastic test materials, together with quality-by-design frameworks for PET and PP nanoplastics and broader discussions of what fit-for-purpose reference materials must look like for environmental studies [54,55,56,57,58,59,60]. These developments matter directly for drinking-water analytics because recovery experiments, instrument benchmarking, and interlaboratory comparison cannot mature without well-characterized materials that mimic the diversity, aging state, and surface chemistry of environmentally relevant particles [57,58,59,60].

Matrix interference remains a first-order issue even when the target polymer is known. Work on polystyrene and polypropylene nanoplastics in complex environmental matrices has shown how background organic matter and other colloids can distort both isolation and confirmation, which is why clean-water best-practice frameworks and broader analytical reviews repeatedly stress orthogonal confirmation, procedural blanks, and careful interpretation of non-specific particle signals [21,24,28,29,61].

A useful way to think about the problem is that measuring nanoplastic requires three types of confidence at once: confidence that the particles were isolated without major bias; confidence that the detected signal is truly plastic; and confidence that the reported quantity corresponds to the environmental question of interest. Most studies achieve one or two of these, but not all three simultaneously. Optical single-particle methods can offer strong chemical confidence but limited throughput. Thermal methods can provide high confidence in polymer mass but limited particle-level context. Scattering methods can offer high particle-number sensitivity but weak chemical confidence. That is why complementary analytical workflows are so important: they distribute confidence across complementary measurements rather than pretending that a single measurement can do everything.

The practical priorities for the next generation of studies are therefore straightforward, even if they are not easy. Researchers should use contamination-controlled workflows with documented blanks; explicitly declare operational size boundaries; validate recovery under matrix-relevant conditions; report both the strengths and failure modes of their methods; and, whenever possible, pair particle-resolved and mass-resolved measurements. Interlaboratory exercises should be designed around realistic aqueous matrices rather than idealized dispersion alone. If the field does not move in that direction, concentration values will continue to accumulate faster than confidence in those values.

These priorities also matter for regulation. WHO’s drinking-water and exposure assessments both underline that uncertainty in occurrence and exposure is still too high for strong health-risk conclusions [1,2]. Better toxicology alone will not solve that problem if exposure measurements remain unstable. Regulatory readiness depends first on exposure metrology, as limits, monitoring programs, and risk assessments all require defensible, comparable analytical data. The future of nanoplastics in drinking water is therefore not only a question of detecting smaller particles; it is a question of building a measurement system that can survive comparison, replication, and policy use.

## 7. Toward a Fit-for-Purpose Workflow for Drinking-Water Monitoring

If the objective is not only discovery but future surveillance, the field needs a practical analytical architecture that reflects what current methods can do. A useful monitoring workflow should not begin by selecting the most fashionable instrument; it should begin by defining the regulatory or scientific question. If the question is whether a water matrix contains polymer-specific nanoplastic mass at all, thermal analysis after controlled preconcentration may be the appropriate anchor. If the question is whether a specific treatment step changes particle morphology or size distribution, particle-resolved microscopy coupled to chemical confirmation becomes more important. If the question is whether a bottled water product contains packaging-derived nanoscale polymers, a high-sensitivity optical method may be the best first-line tool. The current mistake in the literature is often to force a single analytical platform to answer all these questions at once.

A fit-for-purpose workflow for drinking-water monitoring would likely have four layers. The first is contamination-controlled sampling and preprocessing. Sample containers, transfer materials, filtration hardware, and laboratory air should be explicitly selected and documented. Field blanks and procedural blanks should accompany every batch. Recovery experiments should be performed not only in laboratory water but also in matrix-matched water that represents the ionic strength, organic load, and treatment chemistry of the target system [1,5,6,7]. Without this first layer, downstream analytical sophistication has little practical meaning.

The second layer is a screening stage designed to establish whether a sample contains a nanoscale particulate population worth deeper interrogation. Here, techniques such as NTA, DLS, or rapid optical imaging can have legitimate value, provided that their outputs are treated as provisional descriptors rather than definitive counts of nanoplastic. In many settings, a screening layer can help optimize sample volumes, assess aggregation behavior, and identify which fractions deserve confirmatory analysis. What it cannot do is replace polymer-specific identification [5,9].

The third layer is particle-resolved chemical confirmation. Depending on the research question and available infrastructure, this may involve Raman mapping, SERS, AFM-IR, SEM-Raman, or SRS microscopy [10,12,17,18,25,30,31,32,47]. The point is not that every monitoring laboratory should immediately install all these tools. The point is that at least one confirmatory method capable of assigning a plastic identity at the particle scale is needed if the result is to be interpreted as direct evidence of nanoplastic occurrence. In this layer, throughput may be lower, but analytical confidence rises sharply.

The fourth layer is mass-resolved polymer quantification. Py-GC/MS remains particularly valuable here because it provides polymer-specific concentration data that can support temporal trend analyses, source apportionment hypotheses, and cross-matrix comparisons [11,16]. When particle-resolved methods and polymer-mass methods are applied to matched aliquots or to sequential fractions from the same sample, the resulting evidence is much more resilient than evidence from either class alone. For surveillance purposes, this layered strategy may ultimately prove more realistic than any attempt to identify a single universal instrument.

A tiered architecture also makes it easier to integrate emerging methods without sacrificing compatibility. O-PTIR, advanced AFM-IR, AF4-coupled thermal analysis, and future size-resolved hybrid workflows can be positioned as second-line or confirmatory tools rather than as isolated demonstrations [13,37,38,39,40,42,43,51]. The key is that each added method should address a clearly defined analytical gap—particle identity, size-resolved composition, mass concentration, or matrix-specific interference—within a standardized QA/QC framework [24,28,29,57,58,59,60].

A robust reporting framework should accompany this analytical architecture. At minimum, studies should declare the operational definition of nanoplastics used; sample volume; the size range targeted by preconcentration; whether values represent counts, mass, or both; blank-correction procedures; recovery design; the route of polymer confirmation; spectral-matching or marker-ion criteria; LOD/LOQ; and whether reported concentrations apply to individual polymers, pooled plastics, or mixed colloidal fractions. The reporting minima summarized in Table 2 should be treated as analytical validity criteria rather than optional descriptive details. Currently, the literature often omits one or more of them, which is one reason numerical values remain difficult to compare directly [5,6,7,14,15].

A practical reporting template should therefore include three separate validation statements: recovery/trueness for the preparation chain, calibration/LOD/LOQ for the quantitative endpoint, and classification uncertainty for the polymer-identification endpoint. These statements should not be merged. A study can have excellent particle-level identification but weak mass calibration, or strong mass quantification but no particle morphology. Making this separation explicit prevents one validated component from being overextended to the entire workflow.

For drinking-water authorities and environmental monitoring programs, the implication is clear. Near-term surveillance will probably need tiered rather than single-step strategies. Screening laboratories may generate early-warning information, while a smaller number of reference laboratories provide confirmatory particle-level and polymer-level analyses using more advanced instrumentation. This model is already common in other analytical fields where the analyte is difficult to obtain, rare, or method-sensitive. Nanoplastics in drinking water almost certainly belong in that category.

This proposed workflow also has a strategic advantage for research. It makes room for innovation without sacrificing comparability. New methods can be inserted into the layered architecture and judged not by whether they replace every other method, but by which uncertainty they reduce most effectively—size reach, chemical specificity, throughput, matrix tolerance, or quantification. That is a much more mature innovation culture than the current tendency to present each new method as a stand-alone solution.

## 8. Search Auditability and Corpus Structure

Search transparency is important in this review because the synthesis combines a strict PRISMA-guided corpus with a second ring of contextual and metrological literature. The formal corpus was closed on 11 April 2026 and was built from four complementary query families. Table 4 shows how the search was intentionally partitioned: Q1 captured direct drinking-water occurrence; Q2 targeted potable-water and treatment-plant analytical workflows; Q3 extended the search to transferable water matrices; and Q4 captured emerging single-particle and hybrid platforms. This design prevents a narrow drinking-water search from missing methods developed in freshwater, saltwater, or model aqueous systems while keeping the occurrence corpus conservative.

Supplementary File S1 expands this audit into a submission-ready PRISMA package: it reports the PRISMA 2020 checklist, compact and database-compatible Boolean strings, information sources, search-closure date, inclusion and exclusion rules, record-level screening decisions, and the data-extraction fields used for method-centered appraisal. This supporting file is intended to make the review reproducible without relying on unverifiable subscription-gated export counts.

The screening outcome was then separated into direct and transferable evidence blocks rather than treated as a single undifferentiated list. This distinction is central to the manuscript’s logic: direct studies support interpretation of occurrence and exposure, whereas transferable studies support analytical capability, size reach, matrix tolerance, and orthogonal confirmation.

Table 5 contains eight studies directly relevant to drinking water. These records form the core evidence for occurrence because they address tap water, bottled water, potable water, or drinking-water treatment plants. Their methodological diversity also explains why this review avoids pooled concentration estimates: the corpus includes sequential fractionation, SERS, SRS microscopy, AFM-IR, O-PTIR, multimethod comparison, and Py-GC/MS, all of which yield distinct analytical endpoints.

Table 6 contains fourteen studies retained not because they directly measured consumer drinking water, but because they solve analytical problems that drinking-water studies must also solve. These include nanoscale Raman visualization, SERS sensitivity, matrix-dependent detection, separation-linked identification, correlative SEM-Raman analysis, machine-learning-assisted polymer identification, and AI-assisted in situ characterization. Including this block keeps the review technically current without inflating the number of direct occurrence studies.

Table 7 documents the conservative boundary of the synthesis. Guidance reports, narrative reviews, microplastics-only surveys, and surrogate-removal experiments were not counted as primary analytical evidence, even when they were useful for context. This exclusion strategy reduces breadth but improves interpretive discipline: occurrence claims in the review are supported only by studies that analytically addressed nanoplastics in drinking-water-relevant or directly transferable water matrices. Taken together, Table 4, Table 5, Table 6 and Table 7 make the evidence architecture auditable: Table 4 defines how the search was opened; Figure 1 reports how records flowed through screening; Table 5 and Table 6 explain the two evidence blocks; and Table 7 shows why excluded records were not used to support occurrence claims.

## 9. Cross-Study QA/QC, Analytical Performance, Matrix Interference, and Validation Synthesis

The preceding sections describe analytical platforms and matrix-specific evidence. The present section adds the comparative layer needed to evaluate reliability across the included corpus. It should be read as a metrological synthesis rather than as a prevalence estimate: the purpose is to determine which analytical claims are supported by blanks, recovery/trueness evidence, calibration, LOD/LOQ reporting, and orthogonal confirmation.

### 9.1. Study-Level QA/QC and Validation Features

Table 8 directly compares the 22 studies included in the qualitative synthesis. The table distinguishes direct drinking-water evidence from transferable water-matrix method evidence and records whether the reliability of each study rests primarily on particle-resolved identification, polymer-specific mass quantification, matrix testing, or method proof of concept. NR means not reported, not recoverable from the reviewed analytical description, or not applicable to the endpoint; it is not used as a punitive label but as an audit marker.

Table 8 shows that the strongest evidentiary designs are not necessarily those with the smallest nominal size reach, but those that combine contamination control, chemically specific confirmation, and a quantitative endpoint that matches the conclusion. Studies based on Py-GC/MS are stronger for polymer mass but weaker for particle morphology; Raman/SERS studies are stronger for particle-level chemistry but require stricter control of optical artifacts and calibration; NTA/DLS-type outputs are useful only when interpreted as screening descriptors. The table therefore converts method heterogeneity from a narrative limitation into an explicit evidence-quality finding.

### 9.2. Analytical Performance, Reproducibility, and Robustness by Method Family

Table 9 addresses the method-performance gap by separating sensitivity, selectivity, quantification, reproducibility, robustness, and workflow role. This separation is essential because a method may be highly sensitive but poorly standardized, chemically specific but low-throughput, or quantitatively strong while destroying particle-level information.

The central implication of Table 9 is that analytical applicability cannot be inferred from nominal size reach alone. For example, SERS may detect smaller particles than conventional Raman, but the gain in sensitivity is meaningful only if substrate reproducibility, matrix-matched calibration, and blank correction are documented. Conversely, Py-GC/MS may provide the most defensible mass concentration, but it cannot answer particle-number or morphology questions. Method robustness must therefore be judged against the endpoint claimed, not against a generic notion of detection capability.

### 9.3. Matrix Interference and False-Positive Vulnerability

**Figure 4:**
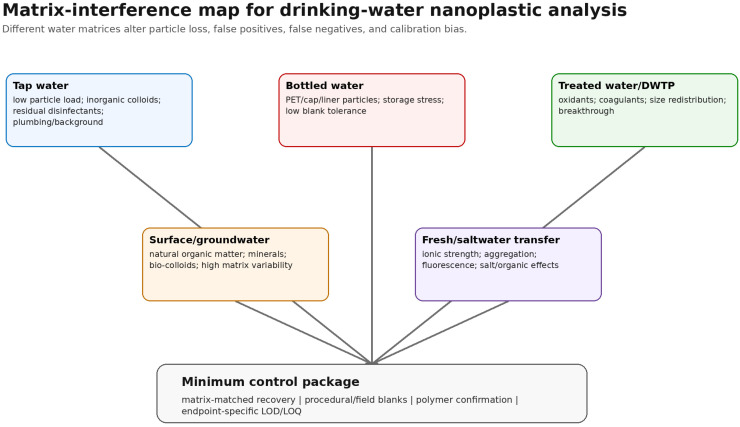
Original matrix-interference map for nanoplastic analysis in drinking-water-related matrices. The figure summarizes the main sources of false positives, false negatives, particle loss, and calibration bias in tap water, bottled water, treated water, source waters, and transfer matrices. The diagram is an original synthesis based on the reviewed literature and is not reproduced from any cited article.

Table 10 and Figure 4 systematize matrix interference across tap water, bottled water, treated water, environmental source waters, and model aqueous systems. The same detector can have very different reliability profiles in these matrices because blank burden, colloid co-enrichment, fluorescence, packaging-derived material, ionic strength, and treatment residuals alter both recovery and classification.

Table 10 shows why a result obtained in model water cannot be transferred automatically to tap, bottled, or treated water. Bottled water is analytically special because packaging-compatible polymer signals may be exposure-relevant while still being difficult to apportion to a specific supply-chain stage. Treated water is equally complex because the measured endpoint may change across treatment stages; a plant can remove large particles while altering, mobilizing, or generating smaller fragments. These matrix-specific mechanisms explain why future studies should report both water chemistry and method-specific matrix tolerance.

### 9.4. Descriptive Heterogeneity and Framework for Future Comparability

The heterogeneity of the present corpus can be characterized descriptively even though a formal meta-analysis remains inappropriate. Table 11 identifies the domains that prevent pooling and defines the minimum reporting elements required for future comparability. This is a more defensible approach than calculating a pooled concentration from non-equivalent endpoints.

The most important heterogeneity is endpoint heterogeneity. A polymer-specific mass concentration, a particle count, a hydrodynamic diameter distribution, and a spectral classification rate are analytically different quantities. Treating them as exchangeable would produce pseudo-precision. A future quantitative synthesis will become possible only when studies report harmonized size windows, polymer-specific endpoints, matrix-matched recovery, and endpoint-specific LOD/LOQ values.

### 9.5. Recovery, Trueness, and Mass-Calibration Status

Recovery and trueness deserve explicit treatment because they determine whether an analytical workflow measures environmental nanoplastics or only the fraction that survives the method. Table 12 summarizes the validation and calibration status of the reviewed corpus at the level of endpoint classes. The counts are conservative because a study was credited only when the design clearly supported the stated category.

Table 12 clarifies two practical conclusions. First, polymer-specific mass quantification is currently more mature in thermal workflows than in optical workflows, but it must be accompanied by recovery and blank correction to support trueness. Second, true metrological confidence is highest when mass-resolved and particle-resolved evidence are generated from the same sample, matched aliquots, or well-defined sequential fractions. This combined design remains uncommon and should be a priority for future drinking-water studies.

### 9.6. Positioning Relative to Recent Reviews

Recent reviews have provided valuable syntheses of detection principles, instrumental advances, and environmental analytical challenges. The present review differs in scope and intent: it treats drinking-water nanoplastics as a metrological measurement-chain problem and evaluates whether available methods support comparable exposure-relevant endpoints. Table 13 makes this positioning explicit.

This comparison does not diminish the contribution of previous reviews; rather, it clarifies the added value of the present manuscript. The distinctive contribution is the integration of PRISMA auditability with study-level QA/QC appraisal, endpoint non-equivalence, recovery/trueness, mass-calibration logic, matrix interference, and a tiered monitoring architecture specifically for drinking-water exposure.

## 10. Limitations of the Current Evidence Base

This review has focused on analytical comparability rather than on pooled concentration estimates because the underlying literature remains too heterogeneous for a statistically meaningful synthesis. Definitions of nanoplastics still vary, operational cutoffs are inconsistent, and many studies do not report the same endpoint. In addition, the field is still so sparse that a small number of influential studies can disproportionately shape perceptions. These limitations do not invalidate literature, but they do mean that conclusions should be weighted toward methodological capability and confidence rather than toward apparent numerical consensus [1,2,5,6,7,14,15].

A related limitation is that many methods have been demonstrated most convincingly either in spiked samples or in relatively simple water matrices. Transfer to real drinking-water conditions remains one of the field’s hardest tests, and the studies that attempt it often do so with small sample numbers, narrowly defined polymer targets, or controlled proof-of-concept conditions [8,9,10,11,12,13,16,17,18,36,47,52,62]. For that reason, current data are best interpreted as evidence of a plausible and increasingly credible occurrence, not as a completed exposure map.

Another limitation is the search architecture itself. The closed formal search relied on publicly accessible scholarly records and backward citation chaining rather than on subscription-only export tools from all bibliographic databases. That decision improved auditability and prevented the reporting of unverifiable database counts, but it may have missed records indexed only in paywalled discovery platforms. The search auditability section should therefore be read as transparent and frozen, not as a claim of impossible exhaustiveness.

## 11. Conclusions

The literature now supports a firm but cautious conclusion: nanoplastics have been reported in tap water, bottled water, potable water, and drinking-water treatment systems, but the certainty of those measurements still depends heavily on the design of the analytical workflow. The field has demonstrated detection, yet it has not achieved metrological closure.

The main scientific problem is not a lack of potentially useful instruments. It is the absence of a harmonized, end-to-end measurement workflow that can reproducibly connect sample preparation, particle identification, chemical confirmation, and quantitative reporting. No single current technique can simultaneously deliver reliable size reach, particle-resolved morphology, polymer-specific chemistry, and concentration metrics in complex drinking-water matrices. Conventional FTIR is too coarse for true nanoscale work; Raman-based methods remain sensitive to matrix effects and have throughput limitations; scattering methods lack intrinsic chemical specificity; and Py-GC/MS sacrifices particle-level context in exchange for strong polymer mass information.

The most defensible way forward is therefore orthogonal. Contamination-controlled preconcentration and separation should be coupled to particle-resolved chemical confirmation and polymer-specific mass analysis wherever feasible. Studies should report what their methods can and cannot measure, instead of translating one type of analytical output into broader claims than the data support. Reference materials, benchmark protocols, and interlaboratory exercises are not optional refinements; they are prerequisites for a mature field.

Potable-water exposure is the downstream analytical endpoint of a connected polymer-particle system. As plastic contamination moves through source waters, treatment systems, packaging infrastructure, and laboratory workflows, it becomes a molecular and particulate signal that current analytical science can only partially resolve. Closing that measurement gap now requires traceable chemical identification, validated quantitative endpoints, harmonized QA/QC, and interlaboratory comparability.

## Figures and Tables

**Table 1:** Comparative assessment of current analytical platforms for nanoplastics in drinking-water-related matrices.

Method Family	Demonstrated Size Reach in Water Studies	Primary Analytical Output	Main Strength	Main Limitation	Key Refs
Raman mapping/microscopy	Down to ~100 nm under controlled conditions	Particle-resolved chemical fingerprints	Non-destructive polymer identification	Low throughput; fluorescence and matrix effects	[17,25,27,28]
SERS	Down to ~20–100 nm in recent aqueous studies	Enhanced particle-level chemical signal	High sensitivity in water and packaging-relevant matrices	Substrate dependence; calibration and reproducibility challenges	[10,27,30,31,32,35]
AFM-IR	20–1000 nm	Single-particle IR-based chemical identification	Nanoscale chemical specificity with morphology context	Specialized instrumentation and very low throughput	[12,42,43]
O-PTIR/submicron infrared spectroscopy	Submicron to nanoscale-associated domains in recent water and environmental studies	Infrared chemical spectrum with improved spatial precision	Bridges IR chemical specificity and submicron targeting; complementary to AFM-IR	Still emerging for complex waters; throughput and quantitative standardization remain limited	[13,40,41,42,43]
NTA/DLS	Colloidal and nanoscale populations	Hydrodynamic size distributions and count proxies	Useful screening and aggregation assessment	No intrinsic chemical specificity	[9,44,45,46]
Py-GC/MS	Preconcentrated NP fractions	Polymer-specific mass concentration	Strong quantitative chemical information	Destructive; no particle count or morphology	[11,16,51]
Hybrid platforms (SEM-Raman, SRS)	Single-particle to nanoscale	Integrated imaging plus chemical identification	Best route to orthogonal confirmation	Not yet standardized for routine monitoring	[18,36,46,47,50,51]

Note. Demonstrated size reach refers to what has been reported in the cited water studies, not to the theoretical instrument limit under idealized conditions.

**Table 2:** Critical analytical parameters that should be reported for nanoplastic studies in drinking-water-related matrices.

Parameter	Analytical Chemistry Relevance	Minimum Reporting Expectation	Preferred Implementation
Operational size boundary	Defines which fraction can be called nanoplastic and prevents conflation of colloids, submicron microplastics, and aggregates.	Report nominal cutoff, membrane pore size, or separation window, and lower/upper particle-size boundary.	Use sequential fractionation or size-resolved separation with recovery checks across each fraction.
Sample container and blanks	Background contamination can be comparable to the true signal in low-particulate drinking water.	Report container material, field blanks, procedural blanks, and airborne/background controls.	Use glass or metal contact surfaces where feasible and apply batch-specific blank correction.
Recovery and particle loss	Adsorption onto vessels, filters, and tubing can bias particle counts and mass concentrations.	Report on spike-recovery design and any correction factors.	Use matrix-matched recovery with polymer classes and particle sizes, as in the analytical target.
Pretreatment chemistry	Oxidation, digestion, evaporation, and filtration can alter surface chemistry, aggregation, and polymer signatures.	Report reagents, contact time, temperature, pH, and digestion/fractionation sequence.	Validate that pretreatment removes interfering matter without generating or destroying target polymer signals.
Polymer confirmation route	A nanoscale particle count is not a nanoplastic count unless polymer identity is established.	State whether confirmation is Raman, SERS, AFM-IR, O-PTIR, Py-GC/MS, MALDI-TOF-MS, or hybrid.	Use at least one particle-resolved method and, when possible, one independent polymer-specific chemical or thermal endpoint.
Quantitative endpoint	Particle number, hydrodynamic size, spectral identification, and polymer mass are not interchangeable.	Declare whether results are reported as particles/L, mass/L, size distribution, polymer-specific mass, or qualitative identity.	Report matched endpoints from paired aliquots or sequential fractions to avoid overinterpreting one metric.
LOD, LOQ, and calibration	Claims of trace detection require transparent analytical sensitivity and calibration strategy.	Report LOD/LOQ, calibration material, fitting model, and blank treatment.	Use polymer-specific calibration and reference/test materials with stated size, aging state, and surface chemistry.
Data processing and spectral matching	Automated classification can inflate confidence if thresholds and libraries are not disclosed.	Report library, pre-processing, baseline correction, match threshold, and manual/automated validation rules.	Use open or auditable spectral decision criteria and confirm ambiguous particles by an orthogonal method.
Uncertainty and transferability	Interlaboratory comparability depends on measurement uncertainty rather than instrument novelty.	Report technical replicates, matrix effects, and sources of uncertainty.	Participate in interlaboratory exercises and benchmark against common test materials and shared QA/QC protocols.

Note. Table 2 is intended as a reporting and review checklist, not as a hierarchy of methods. Its purpose is to make explicit which analytical claims are supported by the workflow and which remain inferential.

**Table 3:** Representative studies in drinking-water-related matrices and the main analytical caution attached to each result.

Matrix	Representative Study	Main Analytical Workflow	Principal Finding	Key Caution
Tap water	Li et al., 2022 [8]	Sequential filtration + FTIR/AFM-IR + Py-GC/MS	Reported nanoplastics in the 58–255 nm range and 1.67–2.08 µg/L	The result is tightly linked to operational cutoffs and sample treatment
Bottled drinking water	Huang et al., 2022. [9]	Nanoparticle isolation + NTA + molecular characterization	Detected organic nanoscale particles and suggested bottle degradation as a source	NTA alone does not provide polymer-specific confirmation
Bottled drinking water	Zhang et al., 2023 [10]	SERS-based PET detection	Detected PET nanoplastics with an average size of ~88.2 nm	Packaging-related source plausible, but supply-chain apportionment remains composite
Potable waters	Okoffo & Thomas, 2024 [11]	Pretreatment + Py-GC/MS	Quantified selected polymers in low-µg/L ranges	Mass-based output does not preserve particle size or count
Drinking-water treatment plant	Li et al., 2024. [12]	AFM-IR + Py-GC/MS	Detected PE and PVC nanoplastics and implicated ozonation as a transformation/source zone	Treatment performance depends on the endpoint being evaluated
Treated drinking water and bottled water	Hart & Lenhart, 2026 [13]	Comparative occurrence analysis	Reinforced that NP occurrence remains poorly understood because methods are limited	Interpretation remains constrained by analytical heterogeneity

**Table 4:** Prespecified query families used to close the formal search.

Query Family	Core Search Logic	Purpose
Q1	(nanoplastic* OR nano-plastic*) AND (“drinking water” OR “tap water” OR “bottled water” OR “potable water”) AND (detection OR identification OR quantification)	Direct occurrence in drinking-water-related matrices
Q2	(nanoplastic* OR nano-plastic*) AND (“drinking water treatment plant” OR potable water) AND (“AFM-IR” OR “Py-GC/MS” OR Raman OR SERS OR NTA)	Treatment-plant and potable-water analytical studies
Q3	(nanoplastic* OR nano-plastic*) AND (“surface water” OR groundwater OR freshwater OR saltwater) AND (Raman OR SERS OR “Py-GC/MS” OR microscopy)	Transferable water-matrix analytical methods
Q4	(nanoplastic* OR nano-plastic*) AND water AND (“single-particle” OR “machine learning” OR “SEM-Raman” OR “field-flow fractionation” OR “SRS microscopy” OR holographic)	Hybrid and emerging single-particle platforms

Note. The asterisk (*) is a truncation wildcard used in the search strings to retrieve all word variants sharing the specified stem (e.g., nanoplastic and nanoplastics).

**Table 5:** Direct drinking-water-relevant studies included in the final qualitative synthesis (n = 8).

Study	Matrix/Focus	Main Analytical Workflow	Why Included
Li et al. 2022 [8]	Tap water	Sequential fractionation + FTIR + AFM-IR + Py-GC/MS	First practical occurrence workflow in tap water
Huang et al. 2022 [9]	Bottled drinking water	NTA + AFM imaging + compositional characterization	Packaging-related bottled water nanoparticle evidence
Zhang et al. 2023 [10]	Commercial bottled water	SERS identification of PET nanoplastics	Direct polymer-specific bottled-water identification
Okoffo and Thomas 2024 [11]	Environmental and potable waters	Ultrafiltration/digestion + Py-GC/MS	Polymer-specific mass quantification in potable waters
Li et al. 2024 [12]	Drinking water treatment plant	AFM-IR + Py-GC/MS	Particle-resolved plus mass-resolved DWTP evidence
Qian et al. 2024 [18]	Bottled water	SRS microscopy single-particle imaging	High-sensitivity direct bottled-water particle imaging
Xu et al. 2024 [52]	Drinking water treatment plant	Py-GC/MS across size fractions	Mass-based MP/NP profiles across the full treatment train
Hart and Lenhart 2026 [13]	Treated drinking water vs. bottled water	O-PTIR/multimethod comparison	Most recent direct comparison of treated and bottled waters

**Table 6:** Directly transferable water-matrix method studies included in the final qualitative synthesis (*n* = 14).

Study	Matrix/Focus	Main Analytical Workflow	Why Included
Sobhani et al. 2020 [25]	Water/proof-of-concept	Raman imaging to 100 nm	Foundational nanoscale Raman demonstration
Lv et al. 2020 [30]	Aquatic environments	In situ SERS	Early aqueous SERS feasibility
Chaisrikhwun et al. 2023 [31]	Various aqueous media	Quantitative SERS	Size-independent quantification across water types
Ruan et al. 2024 [32]	Water	Sol-based SERS	Rapid detection down to 20 nm
Xu et al. 2022 [16]	Surface water and groundwater	Py-GC/MS	Transferable mass-based quantification in environmental waters
Li et al. 2022 [47]	Single particles	SEM-Raman	Morphology + chemistry at the single-particle level
Fang et al. 2020 [26]	Raman imaging	Sub-diffraction interpretation	Methodological clarification of Raman limits
Schwaferts et al. 2020 [49]	Aqueous dispersions	FFF-Raman + optical tweezers	Separation linked to identification
Schmidt et al. 2021 [48]	Complex environments	Correlative SEM-Raman	Complex-matrix nanoscale detection
Shorny et al. 2023 [34]	Single particles/agglomerates	SERS imaging	Single-particle identification down to 100 nm
Luo et al. 2023 [33]	Environmental nanoplastics	RSN-array SERS	Sensitive detection with small sample volumes
Wang et al. 2024 [36]	Aquatic systems	AI-assisted nano-DIHM	Real-time in situ physicochemical characterization
Caldwell et al. 2024 [17]	Fresh- and saltwater spikes	Raman spectroscopy	Matrix dependence was directly tested in water
Xie et al. 2023 [62]	Individual nanoplastics	Raman + machine learning	Automated particle-level polymer identification

**Table 7:** Records excluded during the closed formal search (*n* = 11).

Record	Stage	Reason for Exclusion
WHO 2019 [1]. Microplastics in drinking water.	Title/abstract	Guidance report, not primary research
WHO 2022 [2]. Dietary and inhalation exposure to nano- and microplastic particles.	Title/abstract	Guidance report, not primary research
Capodaglio 2025 [63]. Micro- and Nano-Plastics in Drinking Water: Threat or Hype?	Title/abstract	Narrative review/commentary
Zhang et al. 2024 [64]. Microplastics and nanoplastics in drinking water and beverages: occurrence and human exposure.	Title/abstract	Review article
Maharjan 2024/2025 [65]. Microplastic pollution in bottled water: a systematic review.	Title/abstract	Microplastics-focused review
Gambino et al. 2022 [66]. Occurrence of Microplastics in Tap and Bottled Water.	Title/abstract	Review article; no nanoplastic analytical synthesis
Romphophak et al. 2024 [67]. Removal of microplastics and nanoplastics in water treatment systems.	Title/abstract	Broad treatment review; not analytical detection
Binelli et al. 2026 [68]. From Aquifer to Tap: plastic particles along a drinking-water supply chain.	Title/abstract	Plastic-particle monitoring without nanoplastic analytical focus
Pulido-Reyes et al. 2022 [53]. Nanoplastics removal during drinking water treatment.	Full text	Surrogate Pd-labeled removal study; not environmental NP detection
Zhang et al. 2020 [69]. Removal efficiency of micro- and nanoplastics during drinking water treatment.	Full text	Engineered-particle removal study; not analytical occurrence/detection
Sefiloglu et al. 2025 [70]. Quantitative analysis of microplastics from source to tap.	Full text	Microplastics-only monitoring without nanoplastic detection

**Table 8:** Study-level QA/QC and validation features across the included qualitative corpus (n = 22).

Study	Evidence Block/Matrix	Blank and Contamination Controls	Recovery/Trueness Evidence	Calibration/LOD-LOQ Status	Endpoint and Confirmation Route	Main Reliability Implication
Li et al. 2022 [8]	Direct/tap water	Partial: contamination context considered but not harmonized across laboratories	Partial: fractionation-based workflow; full trueness remains operational-cutoff dependent	Partial: mass and particle endpoints are not directly interchangeable	Sequential fractionation + FTIR/AFM-IR + Py-GC/MS	Strong orthogonality, but concentration depends on filtration cutoffs and particle losses
Huang et al. 2022 [9]	Direct/bottled water	Partial: low-particle matrix requires stringent blank interpretation	NR or limited for polymer-specific recovery	Not a polymer-mass calibration study	NTA + AFM imaging + compositional characterization	Good nanoparticle evidence; NTA alone cannot prove plastic identity
Zhang et al. 2023 [10]	Direct/bottled water	Partial: packaging matrix considered; blank burden remains decisive	Partial: SERS transfer to bottled water; recovery not equivalent to trueness	SERS calibration is substrate- and polymer-dependent	SERS identification of PET NPs	High PET specificity, but source attribution and interlaboratory comparability remain constrained
Okoffo and Thomas 2024 [11]	Direct/potable and environmental waters	Reported within Py-GC/MS workflow; blank correction is central	More suitable for recovery assessment than imaging-only methods	Calibration-based polymer-mass endpoint; LOD/LOQ should be polymer-specific	Ultrafiltration/digestion + Py-GC/MS	Strong for polymer mass; no particle count or morphology after pyrolysis
Li et al. 2024 [12]	Direct/DWTP	Reported in a treatment-train context; contamination control remains essential	Partial to strong: paired particle-resolved and mass-resolved workflow strengthens trueness interpretation	Py-GC/MS component supports mass calibration; AFM-IR supports identity	AFM-IR + Py-GC/MS	Best direct example of orthogonal confidence, but throughput is low
Qian et al. 2024 [18]	Direct/bottled water	Requires strict blank and imaging-control context	NR for classical recovery/trueness as mass endpoint	Not primarily a bulk mass-calibration study	SRS single-particle chemical imaging	High-speed particle-resolved imaging; regulatory calibration still immature
Xu et al. 2024 [52]	Direct/DWTP	Reported in treatment-train monitoring context	Partial: recovery depends on fractionation and polymer marker stability	Py-GC/MS mass calibration supports quantitative comparison across fractions	Py-GC/MS across size fractions	Useful for mass profiles and removal; particle morphology is lost
Hart and Lenhart 2026 [13]	Direct/treated vs. bottled water	Comparative matrix design makes blank treatment particularly important	Partial/NR: method comparison more than formal trueness study	Depends on O-PTIR/multimethod endpoint; not a universal calibration solution	O-PTIR/multimethod comparison	Highly relevant comparison, but inference remains method-bound
Sobhani et al. 2020 [25]	Transferable/water proof-of-concept	Controlled laboratory context	NR for environmental matrix trueness	No routine LOD/LOQ framework for field monitoring	Raman imaging to 100 nm	Foundational size-reach evidence; transfer requires matrix validation
Lv et al. 2020 [30]	Transferable/aquatic environments	Partial; SERS controls depend on substrate and deposition route	Limited for classical recovery across matrices	SERS sensitivity reported, but comparability is substrate-specific	In situ SERS	Useful feasibility; reproducibility depends on SERS substrate standardization
Chaisrikhwun et al. 2023 [31]	Transferable/aqueous media	Controlled aqueous comparisons	Partial: method evaluates aqueous-media effects	Quantitative SERS calibration across size classes	Quantitative SERS	Strong calibration concept; external transfer requires substrate harmonization
Ruan et al. 2024 [32]	Transferable/water	Controlled water experiments	NR/partial for real-matrix recovery	Sensitivity demonstrated down to 20 nm; LOD/LOQ context must be substrate-specific	Sol-based SERS	Excellent size reach; matrix and substrate effects limit routine deployment
Xu et al. 2022 [16]	Transferable/surface and groundwater	Py-GC/MS workflow requires blank correction	More amenable to recovery assessment than particle-only methods	Calibration-based polymer-mass quantification	Ultrafiltration/digestion + Py-GC/MS	Strong transferable mass endpoint; no particle morphology
Li et al. 2022 [47]	Transferable/single particles	Controlled single-particle context	NR for water-matrix recovery	Not a bulk LOD/LOQ mass method	SEM-Raman	Good morphology-chemistry linkage; low throughput
Fang et al. 2020 [26]	Transferable/Raman method	Controlled imaging context	NR for environmental trueness	Performance relates to optical resolution and signal interpretation	Sub-diffraction Raman interpretation	Clarifies optical limits; not a monitoring calibration study
Schwaferts et al. 2020 [49]	Transferable/aqueous dispersion	Controlled separation-analysis workflow	Partial: separation step can support recovery assessment	Not a universal mass-calibration framework	FFF-Raman + optical tweezers	Strong size-separation logic; specialized implementation
Schmidt et al. 2021 [48]	Transferable/complex matrices	Complex-matrix controls required	NR or partial for quantitative trueness	Not primarily a LOD/LOQ mass endpoint	Correlative SEM-Raman	Good confirmatory value; throughput and preparation remain limiting
Shorny et al. 2023 [34]	Transferable/single particles/agglomerates	Controlled SERS imaging context	NR for water-matrix recovery	SERS performance depends on substrate and agglomeration state	SERS imaging	Single-particle identification possible; reproducibility requires substrate control
Luo et al. 2023 [33]	Transferable/environmental NPs	Controlled small-volume SERS platform	Partial/NR for full environmental recovery	Sensitivity depends on ring-shaped nanogap substrate	RSN-array SERS	High sensitivity; substrate fabrication controls comparability
Wang et al. 2024 [36]	Transferable/aquatic systems	AI-assisted workflow requires imaging and classification controls	NR for classical recovery/trueness	Not a polymer-mass calibration method	AI-assisted nano-DIHM	High-throughput potential; chemical specificity must be verified
Caldwell et al. 2024 [17]	Transferable/fresh- and saltwater spikes	Spiked matrix design directly tests matrix effects	Partial: matrix-dependent detectability evaluated	Not a polymer-mass LOD/LOQ framework	Raman spectroscopy	Key evidence that water matrix changes Raman detection reliability
Xie et al. 2023 [62]	Transferable/individual particles	Controlled spectral classification context	NR for environmental recovery	Machine-learning validation rather than mass calibration	Raman + machine learning	Automated classification helps, but training-set and spectral-threshold bias remain

Note. NR = not reported, not recoverable from the reviewed methodological description, or not applicable to the stated endpoint. The table evaluates reporting and metrological interpretability, not the overall scientific merit of individual studies.

**Table 9:** Comparative analytical-performance matrix for nanoplastic methods in drinking-water-related workflows.

Method Family	Sensitivity/Size Reach	Chemical Specificity and Selectivity	Quantification and LOD/LOQ Interpretability	Reproducibility and Robustness Risks	Best-Fit Role in a Monitoring Workflow
Raman mapping/microscopy	Can reach ~100 nm under optimized conditions; lower practical limit is matrix- and signal-dependent	High when spectra are clean and library matching is controlled	LOD/LOQ are not easily portable because signal depends on particle position, focus, matrix and thresholding	Fluorescence, weak scattering, laser spot effects, baseline correction and library choice reduce robustness	Confirmatory particle-level identification in selected fractions
SERS	Highest optical sensitivity among Raman-derived approaches; reports down to ~20–100 nm in water studies	Potentially high, but spectra depend on substrate-particle contact and plasmonic hot spots	Quantification possible only with substrate-specific and polymer-specific calibration; LOD/LOQ are not universal	Substrate batch, aggregation, deposition pattern, enhancement heterogeneity and matrix adsorption limit reproducibility	High-sensitivity detection for targeted polymers, especially packaging-relevant matrices
AFM-IR/O-PTIR	Nanoscale or submicron chemical interrogation, depending on platform and sample preparation	High IR-based polymer specificity when particles are accessible and cleanly deposited	Quantitative use remains specialized; LOD/LOQ and throughput are not yet routine for surveillance	Low throughput, demanding deposition, surface effects and operator specialization	Reference-level confirmation of ambiguous particles
NTA/DLS/rapid scattering	Sensitive to nanoscale colloidal populations	Low: no intrinsic polymer identification	Counts and hydrodynamic size are not polymer-specific; LOD/LOQ do not equal nanoplastic detection limits	Aggregation, refractive-index assumptions, non-plastic colloids and salt/organic matter bias outputs	Screening, aggregation assessment and optimization of fractionation
Py-GC/MS	Works after preconcentration; sensitivity depends on polymer marker, sample mass and blank correction	High for selected polymers when marker ions are specific	Best current route for polymer-specific mass; requires calibration, blanks, recovery and polymer-specific LOD/LOQ	Destructive; marker interference and digestion/fractionation losses influence trueness	Mass-resolved polymer quantification and trend monitoring
Hybrid platforms	Can combine nanoscale reach with particle-level context or size separation	Highest when independent modalities converge	Quantification depends on the weakest linked module; inter-module calibration is still developing	Complex instrumentation and data fusion may reduce routine transferability	Reference workflows and interlaboratory benchmarking

**Table 10:** Matrix-interference comparison for drinking-water nanoplastic workflows.

Matrix Class	Dominant Interferents or Bias Sources	Methods Most Affected	Main False-Signal Pathway	Minimum QA/QC Response
Tap water	Low particle burden, inorganic colloids, residual disinfectants, plumbing-derived material and laboratory background	Raman, SERS, NTA/DLS and low-mass Py-GC/MS	Blank signal may be comparable to true signal; small colloids can be misclassified as plastic without polymer confirmation	Field/procedural blanks, glass/metal contact surfaces, matrix-matched recovery and orthogonal identity confirmation
Bottled water	PET/cap/liner particles, transport and storage effects, packaging-derived oligomers and very low blank tolerance	SERS, SRS, O-PTIR and particle counting	Packaging-compatible polymer signals can be real but source apportionment is composite	Batch-specific blanks, unopened-bottle controls, packaging material reference spectra and consumer-chain metadata
Treated water/DWTP	Oxidants, coagulants, filtration residues, shear, transformation and breakthrough of smaller fractions	Py-GC/MS, AFM-IR/O-PTIR and SERS	Removal can appear high by one endpoint but weak by another if size distribution shifts	Paired influent/effluent fractions, polymer-mass endpoint, particle-resolved confirmation and process-stage blanks
Surface water/groundwater	Natural organic matter, minerals, bio-colloids, variable ionic strength and environmental aging	Raman, NTA/DLS, SERS and filtration-based enrichment	Fluorescence, colloid co-enrichment and adsorption losses bias abundance or size estimates	Matrix-matched spikes, digestion validation, recovery checks and independent thermal or IR confirmation
Freshwater/saltwater transfer matrices	Salt effects, aggregation, spectral background, particle-surface interactions and changing refractive index	Raman/SERS, NTA/DLS and optical imaging	Method performance in pure water may not transfer to ionic or organic-rich water	Report water chemistry, test multiple matrices and avoid pooling endpoints without harmonization
Model laboratory water	Artificially low complexity and ideal dispersion	All methods during validation	Performance appears stronger than in real matrices; recovery and LOD may be over-optimistic	Use only as a first validation layer, followed by real-matrix challenge tests

**Table 11:** Descriptive heterogeneity framework for the reviewed nanoplastic evidence base.

Heterogeneity Domain	Observed Variation in the Reviewed Corpus	Why Pooling Is Not Defensible Yet	Minimum Future Comparability Requirement
Matrix	Tap, bottled, potable, DWTP, surface, groundwater, freshwater, saltwater and model aqueous systems	Matrix effects alter recovery, fluorescence, aggregation and blank burden	Report water chemistry and analyze comparable matrix classes separately
Operational size window	Cutoffs range from tens of nanometers to submicron or 20–1000 nm fractions	Different cutoffs describe different particle populations	Declare lower and upper size boundaries and separation efficiency
Endpoint	Particle count, mean size, hydrodynamic size, polymer fingerprint and polymer mass	These are not effect-size equivalents	Report endpoint-specific units without conversion unless empirically justified
Polymer class	PET, PE, PP, PS, PVC and targeted/model polymers occur unevenly	Polymer-specific chemistry affects spectra, pyrolysis markers and recovery	Report polymer-specific results rather than pooled plastic totals only
Sample preparation	Filtration, ultrafiltration, digestion, centrifugation, deposition and evaporation are mixed	Each step can create losses, aggregation or enrichment bias	Use recovery checks through the full preparation chain
Contamination control	Blank reporting is uneven and not yet harmonized	Low-concentration drinking water is highly blank-sensitive	Report field, procedural, airborne and instrument blanks with correction rules
Recovery/trueness	Often partial, model-based or absent; matrix-matched trueness is rare	Measured concentration may reflect workflow efficiency as much as occurrence	Use matrix-matched spikes and report recovery by size and polymer
LOD/LOQ	Availability and meaning vary across spectroscopy, scattering and mass methods	Optical detectability and mass-detection limits are not comparable quantities	Define method-specific LOD/LOQ tied to the reported endpoint
Statistical synthesis	n = 22, but only 8 direct drinking-water studies and highly divergent endpoints	A pooled numerical estimate would be pseudo-precise	Use structured descriptive synthesis until standardized endpoints exist

**Table 12:** Recovery, trueness, and mass-calibration status in the reviewed corpus.

Validation/Calibration Metric	Conservative Status in the Reviewed Corpus	Representative Studies	Metrological Implication
Direct drinking-water relevance	8 studies directly address tap, bottled, potable or DWTP matrices	[8,9,10,11,12,13,18,52]	Exposure relevance is emerging but still method-dependent
Transferable water-matrix method evidence	14 studies provide aqueous method evidence without direct consumer drinking-water occurrence claims	[16,17,25,26,30,31,32,33,34,36,47,48,49,62]	Useful for method capability, but not equivalent to occurrence prevalence
Polymer-specific mass quantification	Concentrated in Py-GC/MS workflows; approximately four included studies provide this class of endpoint	[11,12,16,52]	Mass is the most regulation-friendly endpoint, but particle count/morphology are lost
Particle-resolved plus mass-resolved orthogonality	Rare; strongest direct examples combine AFM-IR or FTIR/AFM-IR with Py-GC/MS	[8,12]	This is the current best route to trueness because identity and mass are cross-checked
Matrix-effect or matrix-transfer testing	Present in selected studies, especially Raman/SERS aqueous-media work and treated-vs-bottled comparison	[13,17,31]	Supports transferability assessment but does not replace full recovery validation
Quantitative SERS calibration	Developing; promising but strongly substrate- and polymer-dependent	[31,32,33]	Calibration must be reported with substrate, deposition and polymer reference conditions
Fully harmonized interlaboratory uncertainty	Not achieved in the drinking-water nanoplastics corpus	[5,6,7,24,57,58,59,60]	Reference materials and interlaboratory exercises are prerequisites for regulatory readiness

**Table 13:** Positioning of the present PRISMA-guided review relative to recent review literature.

Review/Source	Primary Scope	Relationship to the Present Review	Distinct Contribution of the Present Manuscript
Fang et al. 2023 [27]	Analytical options, imaging advances and detection challenges	Provides broad instrumental context for micro/nanoplastic analysis	This review narrows the problem to drinking-water exposure and method traceability
Choi et al. 2024 [28]	Recent analytical advances and detection challenges	Useful overview of emerging technologies	This review evaluates whether those technologies support comparable drinking-water endpoints
Santos et al. 2025 [35]	Comprehensive detection-technique review	Broad technique-centered synthesis	This review adds PRISMA-guided corpus structure, QA/QC extraction and metrological interpretation
Wang et al. 2026 [37]	Waterborne micro/nanoplastics, analytical advances, modellingand future directions	Closest in waterborne scope	This review emphasizes drinking-water matrices, regulatory monitoring layers and method-bound evidence
Zhang et al. 2026 [38]	Advancements and challenges in nanoplastics detection	Recent nanoplastic detection review	This review positions detection limits within recovery, trueness, calibration and endpoint comparability
Kaur et al. 2026 [39]	Physical-chemistry lens on detection principles and limitations	Deep mechanistic discussion of detection techniques	This review translates those limitations into a PRISMA audit and drinking-water QA/QC roadmap
Present review	Drinking-water nanoplastics as metrological and molecular-identification problem	Complements technique-centered reviews	Adds study-level QA/QC comparison, endpoint heterogeneity framework, recovery/trueness discussion and fit-for-purpose monitoring architecture

## Data Availability

No new experimental datasets were generated for this review. The PRISMA checklist, complete search strategy, screening audit log, and data-extraction fields are provided in Supplementary File S1; all records used for the synthesis are cited in the reference list.

## Supplementary Materials

The following supporting information can be downloaded at: https://www.mdpi.com/article/10.3390/molecules31152675/s1, Supplementary File S1: PRISMA 2020 checklist; Table S1: Complete search strategy and query families; Table S2: Eligibility criteria; Table S3: Data-extraction fields; Table S4: Screening log; Table S5: Excluded-record log with reasons for exclusion; and Table S6: Reference-normalization audit; Table S7: Expanded study-level QA/QC and validation extraction matrix used in the revised manuscript; Table S8: Heterogeneity and comparability coding framework added during revision; Table S9. Recovery/trueness and calibration-status summary used to support the revised metrological discussion. References [5,6,7,8,9,10,11,12,13,16,17,18,24,25,26,27,28,29,30,31,32,33,34,36,47,48,49,52,57,58,59,60,62] are cited in Supplementary File S1.
