# Supplementary material for: From Aquatic Pollution to Drinking-Water Exposure: Analytical Challenges in Detecting Nanoplastics in Drinking Water—A PRISMA-Guided Review
Source: Molecules. 2026 Jul 31;31(15):2675. doi: 10.3390/molecules31152675 (PMC13468429; doi:10.3390/molecules31152675)
Supplement: Supplementary file 1 [file molecules-31-02675-s001.zip › molecules-4393074-supplementary.pdf]

Supplementary File S1

This supplementary file provides the PRISMA 2020 audit trail, search strategy, eligibility criteria, screening log, extraction codebook, and quality-appraisal matrix for the PRISMA-guided review. The formal search was closed on 11 April 2026.

Table S1. PRISMA 2020 checklist mapping

PRISMA item	Operational interpretation in this review	Location	Status
Title	Identify the report as a review guided by PRISMA.	Title page and Abstract	Completed
Abstract	Summarize objectives, sources, inclusion, synthesis, and limits.	Abstract	Completed
Rationale	Explain why the review is needed.	Introduction	Completed
Objectives	State explicit review aims.	Introduction	Completed
Eligibility criteria	Define included and excluded records.	Section 2; Table S3	Completed
Information sources	Identify sources searched and date closed.	Section 2; Table S2	Completed
Search strategy	Provide query families and logic.	Section 8; Table S2	Completed
Selection process	Explain screening and full-text assessment.	Section 2; Table S4	Completed
Data collection process	Describe extraction variables.	Section 2; Table S5	Completed
Data items	List data domains.	Table S5	Completed
Risk of bias/quality appraisal	Describe method-centered appraisal.	Section 2; Table S6	Completed
Effect measures	Clarify that pooled effect measures were not used.	Section 2: Limitations	Not applicable
Synthesis methods	Describe qualitative synthesis and evidence blocks.	Sections 2 and 8	Completed
Reporting bias assessment	State why a formal publication-bias analysis was not applicable.	Limitations	Not applicable
Certainty assessment	Explain the analytical-confidence interpretation.	Sections 5-7	Completed
Study selection results	Provide record counts and exclusions.	Figure 1; Tables 5-7; Table S4	Completed
Study characteristics	Summarize matrices and workflows.	Tables 5 and 6	Completed
Risk of bias in studies	Appraise analytical quality rather than clinical bias.	Section 2; Table S6	Completed
Results of individual studies	Summarize principal findings.	Section 5; Tables 3, 5, 6	Completed
Synthesis results	Provide qualitative synthesis.	Sections 4-7	Completed
Reporting biases	Discuss sparse and method-bound evidence.	Limitations	Completed
Certainty of evidence	State why confidence is method-dependent.	Sections 6-10	Completed
Discussion	Interpret results and limitations.	Sections 6-10	Completed
Registration/protocol	Report registration status.	Table S2 note	Not registered
Support	Report funding.	Funding statement	Completed
Competing interests	Report conflicts.	Conflicts of Interest	Completed
Availability of data/materials	State data availability.	Data Availability Statement; Supplementary File S1	Completed

Table S2. Full search strategy and source logic

Search date closed: 11 April 2026. Formal evidence corpus: publicly accessible scholarly records, PubMed-indexed records, publisher landing pages retrievable by title or DOI, and backward citation chaining from key primary papers. Bibliographic normalization using DOI/publisher metadata was performed after selection and did not alter inclusion counts.

Query family	Exact search logic	Purpose
Q1 direct drinking-water occurrence	(nanoplastic* OR nano-plastic*) AND ("drinking water" OR "tap water" OR "bottled water" OR "potable water") AND (detection OR identification OR quantification)	Captured studies measuring nanoplastics in tap, bottled, potable, or drinking water matrices.
Q2 treatment-plant and potable-water analytics	(nanoplastic* OR nano-plastic*) AND ("drinking water treatment plant" OR potable water) AND ("AFM-IR" OR "Py-GC/MS" OR Raman OR SERS OR NTA)	Captured treatment-train studies and potable-water analytical workflows.
Q3 transferable aqueous-matrix methods	(nanoplastic* OR nano-plastic*) AND ("surface water" OR groundwater OR freshwater OR saltwater) AND (Raman OR SERS OR "Py-GC/MS" OR microscopy)	Captured water-matrix methods that are transferable to drinking-water analytics.
Q4 hybrid and single-particle platforms	(nanoplastic* OR nano-plastic*) AND water AND ("single-particle" OR "machine learning" OR "SEM-Raman" OR "field-flow fractionation" OR "SRS microscopy" OR holographic)	Captured emerging single-particle, hybrid, or data-driven platforms with explicit water relevance.

Table S3. Eligibility criteria

Decision	Criterion
Include	Original peer-reviewed studies describing the detection, identification, or quantification of nanoplastics in tap water, bottled water, potable waters, drinking-water treatment systems, or directly transferable water matrices.
Include	Studies using particle-resolved spectroscopy/microscopy, enhanced Raman methods, AFM-IR, O-PTIR, SRS, Py-GC/MS, or hybrid platforms when the matrix or method had explicit relevance to water.
Exclude	Guidance reports, reviews, editorials, commentaries, and broad policy documents not reporting primary analytical detection.
Exclude	Microplastics-only studies without nanoscale analytical claims.
Exclude	Pure toxicology studies, unless the analytical method itself directly supports nanoplastic detection or identification.
Exclude	Surrogate-removal experiments using engineered labeled particles when no environmental or quasi-environmental nanoplastic detection was performed.

Table S4. Screening log

Screening stage	n	Audit note
Unique records identified	33	Deduplicated by title and DOI.
Excluded at title/abstract screening	8	Reviews, guidance documents, or records outside the nanoscale analytical scope.
Full texts assessed	25	Potentially eligible primary or method papers.
Excluded after full-text assessment	3	Surrogate-removal studies or microplastics-only monitoring without environmental nanoplastic analytical detection.
Included in qualitative synthesis	22	Eight direct drinking-water studies and fourteen directly transferable water-matrix method studies.

Table S5. Data extraction codebook

Extraction field	Definition
Matrix/domain	Tap water, bottled water, potable water, drinking water treatment plant, surface water, groundwater, freshwater, saltwater, or a model aqueous system.
Sample volume and handling	Sample volume, container material, storage, transport, and whether field/procedural blanks were used.
Pretreatment and preconcentration	Filtration, ultrafiltration, centrifugation, digestion, evaporation, deposition, and fractionation cutoffs.
Particle-size boundary	Operational minimum and maximum particle sizes targeted or inferred.
Polymer confirmation route	Raman, SERS, AFM-IR, O-PTIR, SRS, SEM-Raman, Py-GC/MS, MALDI-TOF-MS, or combined methods.
Quantitative endpoint	Particle count, hydrodynamic size, mean size, polymer-specific mass, qualitative identification, or combined endpoint.
Calibration and recovery	LOD/LOQ, calibration materials, recovery experiments, internal standards, and matrix-matched controls when available.
Analytical caution	Main limitation affecting interpretation: matrix interference, low throughput, lack of chemical specificity, destructive measurement, or operational cutoff bias.

Table S6. Method-centered quality-appraisal matrix

Domain	Question	Appraisal logic
Blanks and contamination control	Were field, transport, procedural, and instrument blanks reported and interpreted?	High if blanks were included and used; low if no blank context was reported.
Sample-treatment integrity	Could digestion, filtration, centrifugation, or transfer cause particle loss, aggregation, fragmentation, adsorption, or chemical alteration?	High if recovery/loss was experimentally tested; low if assumed.
Chemical specificity	Was polymer identity confirmed by a chemically specific method rather than by size or scattering alone?	High if particle-resolved or polymer-specific confirmation was used.
Quantitative traceability	Were LOD, LOQ, calibration range, recovery, and uncertainty described for quantitative claims?	High if quantitative controls were reported and matrix-relevant.
Endpoint appropriateness	Did the conclusion match the endpoint measured?	High if particle count, hydrodynamic size, spectral identity, and polymer mass were not treated as interchangeable.

Table S7. Expanded study-level QA/QC and validation extraction matrix used in the revised manuscript

Study	Evidence block / matrix	Blank and contamination controls	Recovery / trueness evidence	Calibration / LOD-LOQ status	Endpoint and confirmation route	Main reliability implication
Li et al. 2022 [8]	Direct / tap water	Partial: contamination context considered but not harmonized across laboratories	Partial: fractionation-based workflow; full trueness remains operational-cutoff dependent	Partial: mass and particle endpoints are not directly interchangeable	Sequential fractionation + FTIR/AFM-IR + Py-GC/MS	Strong orthogonality, but concentration depends on filtration cutoffs and particle losses
Huang et al. 2022 [9]	Direct / bottled water	Partial: low-particle matrix requires stringent blank interpretation	NR or limited for polymer-specific recovery	Not a polymer-mass calibration study	NTA + AFM imaging + compositional characterization	Good nanoparticle evidence; NTA alone cannot prove plastic identity
Zhang et al. 2023 [10]	Direct / bottled water	Partial: packaging matrix considered; blank burden remains decisive	Partial: SERS transfer to bottled water; recovery not equivalent to trueness	SERS calibration is substrate- and polymer-dependent	SERS identification of PET NPs	High PET specificity, but source attribution and interlaboratory comparability remain constrained
Okoffo and Thomas 2024 [11]	Direct / potable and environmental waters	Reported within Py-GC/MS workflow; blank correction is central	More suitable for recovery assessment than imaging-only methods	Calibration-based polymer-mass endpoint; LOD/LOQ should be polymer-specific	Ultrafiltration/digestion + Py-GC/MS	Strong for polymer mass; no particle count or morphology after pyrolysis
Li et al. 2024 [12]	Direct / DWTP	Reported in a treatment-train context; contamination control remains essential	Partial to strong: paired particle-resolved and mass-resolved workflow strengthens trueness interpretation	Py-GC/MS component supports mass calibration; AFM-IR supports identity	AFM-IR + Py-GC/MS	Best direct example of orthogonal confidence, but throughput is low
Qian et al. 2024 [18]	Direct / bottled water	Requires strict blank and imaging-control context	NR for classical recovery/trueness as mass endpoint	Not primarily a bulk mass-calibration study	SRS single-particle chemical imaging	High-speed particle-resolved imaging; regulatory calibration still immature
Xu et al. 2024 [52]	Direct / DWTP	Reported in treatment-train monitoring context	Partial: recovery depends on fractionation and polymer marker stability	Py-GC/MS mass calibration supports quantitative comparison across fractions	Py-GC/MS across size fractions	Useful for mass profiles and removal; particle morphology is lost
Hart and Lenhart 2026 [13]	Direct / treated vs bottled water	Comparative matrix design makes blank treatment particularly important	Partial/NR: method comparison more than formal trueness study	Depends on O-PTIR/multimethod endpoint; not a universal calibration solution	O-PTIR / multimethod comparison	Highly relevant comparison, but inference remains method-bound
Sobhani et al. 2020 [25]	Transferable / water proof-of-concept	Controlled laboratory context	NR for environmental matrix trueness	No routine LOD/LOQ framework for field monitoring	Raman imaging to 100 nm	Foundational size-reach evidence; transfer requires matrix validation

Study	Evidence block / matrix	Blank and contamination controls	Recovery / trueness evidence	Calibration / LOD-LOQ status	Endpoint and confirmation route	Main reliability implication
Lv et al. 2020 [30]	Transferable / aquatic environments	Partial; SERS controls depend on substrate and deposition route	Limited for classical recovery across matrices	SERS sensitivity reported, but comparability is substrate-specific	In situ SERS	Useful feasibility; reproducibility depends on SERS substrate standardization
Chaisrikhwun et al. 2023 [31]	Transferable / aqueous media	Controlled aqueous comparisons	Partial: method evaluates aqueous-media effects	Quantitative SERS calibration across size classes	Quantitative SERS	Strong calibration concept; external transfer requires substrate harmonization
Ruan et al. 2024 [32]	Transferable / water	Controlled water experiments	NR/partial for real-matrix recovery	Sensitivity demonstrated down to 20 nm; LOD/LOQ context must be substrate-specific	Sol-based SERS	Excellent size reach; matrix and substrate effects limit routine deployment
Xu et al. 2022 [16]	Transferable / surface and groundwater	Py-GC/MS workflow requires blank correction	More amenable to recovery assessment than particle-only methods	Calibration-based polymer-mass quantification	Ultrafiltration/digestion + Py-GC/MS	Strong transferable mass endpoint; no particle morphology
Li et al. 2022 [47]	Transferable / single particles	Controlled single-particle context	NR for water-matrix recovery	Not a bulk LOD/LOQ mass method	SEM-Raman	Good morphology-chemistry linkage; low throughput
Fang et al. 2020 [26]	Transferable / Raman method	Controlled imaging context	NR for environmental trueness	Performance relates to optical resolution and signal interpretation	Sub-diffraction Raman interpretation	Clarifies optical limits; not a monitoring calibration study
Schwaferts et al. 2020 [49]	Transferable / aqueous dispersion	Controlled separation-analysis workflow	Partial: separation step can support recovery assessment	Not a universal mass-calibration framework	FFF-Raman + optical tweezers	Strong size-separation logic; specialized implementation
Schmidt et al. 2021 [48]	Transferable / complex matrices	Complex-matrix controls required	NR or partial for quantitative trueness	Not primarily a LOD/LOQ mass endpoint	Correlative SEM-Raman	Good confirmatory value; throughput and preparation remain limiting
Shorny et al. 2023 [34]	Transferable / single particles/agglomerates	Controlled SERS imaging context	NR for water-matrix recovery	SERS performance depends on substrate and agglomeration state	SERS imaging	Single-particle identification possible; reproducibility requires substrate control
Luo et al. 2023 [33]	Transferable / environmental NPs	Controlled small-volume SERS platform	Partial/NR for full environmental recovery	Sensitivity depends on ring-shaped nanogap substrate	RSN-array SERS	High sensitivity; substrate fabrication controls comparability
Wang et al. 2024 [36]	Transferable / aquatic systems	AI-assisted workflow requires imaging and classification controls	NR for classical recovery/trueness	Not a polymer-mass calibration method	AI-assisted nano-DIHM	High-throughput potential; chemical specificity must be verified
Caldwell et al. 2024 [17]	Transferable / fresh- and saltwater spikes	Spiked matrix design directly tests matrix effects	Partial: matrix-dependent detectability evaluated	Not a polymer-mass LOD/LOQ framework	Raman spectroscopy	Key evidence that water matrix changes Raman detection reliability
Xie et al. 2023 [62]	Transferable / individual particles	Controlled spectral classification context	NR for environmental recovery	Machine-learning validation rather than mass calibration	Raman + machine learning	Automated classification helps, but training-set and spectral-threshold bias remain

NR = not reported, not recoverable from the reviewed methodological description, or not applicable to the stated endpoint. The matrix was added to make the reliability appraisal transparent and auditable.

Table S8. Heterogeneity and comparability coding framework added during revision

Heterogeneity domain	Observed variation in the reviewed corpus	Why pooling is not defensible yet	Minimum future comparability requirement
Matrix	Tap, bottled, potable, DWTP, surface, groundwater, freshwater, saltwater and model aqueous systems	Matrix effects alter recovery, fluorescence, aggregation and blank burden	Report water chemistry and analyze comparable matrix classes separately
Operational size window	Cutoffs range from tens of nanometers to submicron or 20-1000 nm fractions	Different cutoffs describe different particle populations	Declare lower and upper size boundaries and separation efficiency
Endpoint	Particle count, mean size, hydrodynamic size, polymer fingerprint and polymer mass	These are not effect-size equivalents	Report endpoint-specific units without conversion unless empirically justified
Polymer class	PET, PE, PP, PS, PVC and targeted/model polymers occur unevenly	Polymer-specific chemistry affects spectra, pyrolysis markers and recovery	Report polymer-specific results rather than pooled plastic totals only
Sample preparation	Filtration, ultrafiltration, digestion, centrifugation, deposition and evaporation are mixed	Each step can create losses, aggregation or enrichment bias	Use recovery checks through the full preparation chain
Contamination control	Blank reporting is uneven and not yet harmonized	Low-concentration drinking water is highly blank-sensitive	Report field, procedural, airborne and instrument blanks with correction rules
Recovery / trueness	Often partial, model-based or absent; matrix-matched trueness is rare	Measured concentration may reflect workflow efficiency as much as occurrence	Use matrix-matched spikes and report recovery by size and polymer
LOD / LOQ	Availability and meaning vary across spectroscopy, scattering and mass methods	Optical detectability and mass-detection limits are not comparable quantities	Define method-specific LOD/LOQ tied to the reported endpoint
Statistical synthesis	n = 22, but only 8 direct drinking-water studies and highly divergent endpoints	A pooled numerical estimate would be pseudo-precise	Use structured descriptive synthesis until standardized endpoints exist

Table S9. Recovery/trueness and calibration-status summary used to support the revised metrological discussion

Validation / calibration metric	Conservative status in the reviewed corpus	Representative studies	Metrological implication
Direct drinking-water relevance	8 studies directly address tap, bottled, potable or DWTP matrices	[8-13,18,52]	Exposure relevance is emerging but still method-dependent
Transferable water-matrix method evidence	14 studies provide aqueous method evidence without direct consumer drinking-water occurrence claims	[16-17,25-32,47-49,62]	Useful for method capability, but not equivalent to occurrence prevalence
Polymer-specific mass quantification	Concentrated in Py-GC/MS workflows; approximately four included studies provide this class of endpoint	[11-12,16,52]	Mass is the most regulation-friendly endpoint, but particle count/morphology are lost
Particle-resolved plus mass-resolved orthogonality	Rare; strongest direct examples combine AFM-IR or FTIR/AFM-IR with Py-GC/MS	[8,12]	This is the current best route to trueness because identity and mass are cross-checked
Matrix-effect or matrix-transfer testing	Present in selected studies, especially Raman/SERS aqueous-media work and treated-vs-bottled comparison	[13,17,28]	Supports transferability assessment but does not replace full recovery validation
Quantitative SERS calibration	Developing; promising but strongly substrate- and polymer-dependent	[28-30]	Calibration must be reported with substrate, deposition and polymer reference conditions
Fully harmonized interlaboratory uncertainty	Not achieved in the drinking-water nanoplastics corpus	[5-7,24,57-60]	Reference materials and interlaboratory exercises are prerequisites for regulatory readiness

Supplementary note on bibliographic normalization

Reference order in the manuscript remains identical to the order of the first citation. The normalization step harmonized author initials, journal abbreviations, year, volume, page range or article number, and DOI format, where the information was available from DOI or publisher records. No new reference was inserted into the main reference sequence.
